# Web-based occupational stress prevention in German micro- and small-sized enterprises – process evaluation results of an implementation study

**DOI:** 10.1186/s12889-024-19102-8

**Published:** 2024-06-17

**Authors:** Miriam Engels, Louisa Scheepers, Judith Engels, Leif Boß, Rebekka Kuhlmann, Johanna Kuske, Lutz Lesener, Valeria Pavlista, Kira Schmidt-Stiedenroth, Mathias Diebig, Sascha A. Ruhle, Florian B. Zapkau, Peter Angerer, Jörg Hoewner, Dirk Lehr, Christian Schwens, Stefan Süß, Ines C. Wulf, Nico Dragano

**Affiliations:** 1 Department of Work and Organisational Psychology, Faculty of Psychology, Open University of the Netherlands, Valkenburgerweg 177, Heerlen, 6419 AT The Netherlands; 2 Institute of Medical Sociology, Centre for Health and Society, Medical Faculty and University Hospital, Heinrich-Heine-University Dusseldorf, Moorenstr. 5, 40225 Düsseldorf, Germany; 3 Institute of Occupational, Social and Environmental Medicine, Centre for Health and Society, Medical Faculty and University Hospital, Heinrich-Heine-University Dusseldorf, Moorenstr. 5, 40225 Düsseldorf, Germany; 4 Chair of Business Administration, in particular Work, Human Resource Management and Organization Studies, Faculty of Business Administration and Economics, Heinrich-Heine-University Dusseldorf, Universitätsstr. 1, 40225 Düsseldorf, Germany; 5 Department of Health Psychology and Applied Biological Psychology, Institute of Psychology, Leuphana University Luneburg, Universitätsallee 1, 21335 Lüneburg, Germany; 6 Chair for Entrepreneurship and Management, Faculty of Management, Economics and Social Sciences, University of Cologne, Albertus‑Magnus‑Platz, 50923 Köln, Germany; 7 K12 Agentur für Kommunikation und Innovation GmbH, Schirmerstr. 76, 40211 Düsseldorf, Germany; 8 Department of Human Resource Studies, Tilburg University, Prof. Cobbenhagenlaan 225, Tilburg, 5037 DB The Netherlands; 9 Institute for International Business, Department of Global Business and Trade, Vienna University of Economics and Business, Welthandelsplatz 1, Wien, 1020 Austria; 10 German Social Accident Insurance Institution for the Administrative Sector, Markgrafenstraße 18, 10969 Berlin, Germany; 11 Department of Work and Organisational Psychology, Faculty I - Psychology, Trier University, Universitätsring 15, 54296 Trier, Germany

**Keywords:** Web-based intervention, Micro- and small-sized enterprises, Occupational health, Psychosocial risk assessment, stress management

## Abstract

**Background:**

Structural and behavioral interventions to manage work-related stress are effective in employees. Nonetheless, they have been implemented insufficiently, particularly in micro- and small-sized enterprises (MSE). Main barriers include a lack of knowledge and limited resources, which could potentially be overcome with simplified web-based alternatives for occupational stress prevention. However, there is a lack of implementation research about web-based prevention in realistic settings of MSE.

**Objective:**

The aim of this study is to evaluate the implementation process and success of an integrated web-based platform for occupational stress prevention (“System P”) and to identify potential barriers for its uptake and use in MSE in Germany.

**Methods:**

This study with a mixed-methods approach investigates eight process-related outcomes in a quantitative part I (adoption, reach, penetration, fidelity/dose, costs, acceptability) and a qualitative part II (acceptability, appropriateness and feasibility). Part I has a pre-post design with two measurements (6 months apart) with 98 individual participants and part II consists of 12 semi-structured interviews with managers and intercorporate stakeholders.

**Results:**

Part I revealed shortcomings in the implementation process. Adoption/Reach: Despite extensive marketing efforts, less than 1% of the contacted MSE responded to the offer of System P. A total of 40 MSE registered, 24 of which, characterized by good psychosocial safety climate, adopted System P. Penetration: Within these 24 MSE, 15% of the employees used the system. Fidelity/Dose: 11 MSE started a psychosocial risk-assessment (PRA), and no MSE finished it. The stress-management training (SMT) was started by 25 users and completed by 8. Costs: The use of System P was free of charge, but the time required to engage with was an indirect cost. Part II added insights on the perception of the web-based intervention: Acceptance of System P by users and stakeholders was good and it was assessed as appropriate for MSE. Results for feasibility were mixed.

**Conclusions:**

Although System P was generally perceived as useful and appropriate, only a small number of contacted MSE implemented it as intended. Prior experience and sensitivity for occupational (stress) prevention were mentioned as key facilitators, while (perceived) indirect costs were a key barrier. Enabling MSE to independently manage stress prevention online did not result in successful implementation. Increasing external support could be a solution.

**⁺ Full project name:**

“PragmatiKK – Pragmatische Lösungen für die Implementation von Maßnahmen zur Stressprävention in Kleinst- und Kleinbetrieben” (= Pragmatic solutions for the implementation of stress prevention interventions in micro and small-sized enterprises).

**Trial registration:**

German Register of Clinical Studies (DRKS) DRKS00026154, date of registration 2021-09-16.

**Supplementary Information:**

The online version contains supplementary material available at 10.1186/s12889-024-19102-8.

## Introduction

### Background

Work-related stress is associated with an increased risk for several severe health problems like cardiovascular diseases and depression in employees [1]. Due to the frequency of its occurrence, it is also a major economic challenge, e.g. regarding the costs of healthcare, sick leave, early retirement or disability [2]. These costs can be especially detrimental to smaller businesses because they lack staff to compensate longer absences. In 2021, the vast majority of all enterprises in Europe were micro- (under ten employees), small- (under 50 employees) and medium (under 250 employees) sized enterprises making up 64% of total employment in Europe. Micro enterprises account for more than 90% of all enterprises in Europe [3, 4]. In Germany, 38% of all employees work in micro- and small-sized enterprises (MSE) with less than 50 employees [5]. Yet, studies on occupational stress prevention in the setting of MSE are very rare.

### Occupational stress prevention

There are two types of interventions intended to reduce work-related stress and thus to prevent (mental) illnesses: (1) structural interventions at an organizational level and (2) behavioral interventions at an individual level. Structural interventions aim to improve working conditions. One of the most important measures at the organizational level is the psychosocial risk-assessment (PRA) at the workplace [6, 7]. Behavioral interventions aim to improve the coping strategies and resilience of employees and include stress-management training (SMT) and other measures at an individual level. Experts recommend a combination of both approaches to prevention in order to sustain mental health at the workplace [8–10].

Reviews of workplace-based interventions to reduce work-related stress and improve (mental) health show clear benefits [11]. General evidence for the effectiveness of workplace-based interventions at organizational level is strong, especially for interventions that aim to increase employee control (as does PRA) [12, 13]. The PRA is a process for assessing and reducing psychosocial risks at work (work processes or work populations), such as high workloads, unfavorable working environments or conflictual interpersonal relationships [14, 15]. In Germany, the process is specified by the Joint German Occupational Safety and Health Strategy [8, 15] and comprises seven steps: (1) preparation of the overall process by defining the area of investigation, (2) measurement of psychosocial stress at work, (3) analysis of psychosocial stress at work, (4) development and implementation of measures, (5) effectiveness monitoring, (6) updating and maintenance of the process and (7) documentation. Moreover, studies on the effectiveness of behavioral interventions, specifically SMT, have shown positive effects on stress and stress-related outcomes [16].

Yet, occupational stress prevention measures are not or only inadequately implemented in many companies [17, 18]. Insufficient dissemination and depth of implementation is particularly prevalent among MSE [19]. In Germany, for example, mental health prevention in the context of occupational health management is often neglected in MSE even though the inclusion of PRA in general risk assessments is legally mandatory since 2013: Most micro (85%) and small (67%) enterprises do not conduct a work place risk assessment including psychosocial factors and only 4% of micro and 7% of small enterprises complete a whole PRA cycle [17].

### Barriers to occupational stress prevention in MSE

Implementation research investigates the real-world application of evidence-based interventions and the factors that might hinder it [20]. In addition to the effectiveness in terms of the desired changes, outcomes related to the process of implementation in a particular setting are equally important for the evaluation of (adapted) interventions [21]; also see Table 1). Implementation research also tries to identify all determinants that help to explain the success or failure of an intervention. According to the Consolidated Framework For Implementation Research (CFIR) [22, 23] barriers can be found in any of the five main domains: (1) innovation (intervention) characteristics, (2) outer setting, (3) inner setting, (4) individuals and (5) the implementation process. Although research on the topic is rather scarce in the setting of MSE (or refers to samples of small- to medium-sized enterprises (SME)), some international studies (e.g. Netherlands, Ireland, Germany) have identified possible barriers in regard to these domains:

1. Regarding the intervention characteristics, Benning et al. [24] reported that the (perceived) complexity of prevention measures is a main barrier to the implementation in MSE. Pavlista et al. [25] found a negative image of PRA among representatives of MSE who also considered it the wrong approach for MSE.
2. With regard to the outer setting, a general lack of resources, specifically a shortage of staff and limited financial resources, has been reported to be one of the most important barriers to implement occupational health measures (including mandatory risk assessments for physical risks at the workplace) in SME and MSE [24]. Resource constraint is one of the typical characteristics of SME and likely to be even more prominent in micro-sized enterprises [26]. It has been argued that due to limited financial or human resources, the room for maneuver of MSE is often restricted so that they focus more on securing their existence and managing their day-to-day business instead of practicing occupational health management [27]. In addition, barriers to the implementation of PRA in MSE include ignorance of the legal obligation to conduct a PRA, not understanding the necessity of the assessment and an assumption of lack of acceptance by employees [25]. Managers of MSE may prefer to deal with work-related stress in an informal manner, outside the framework of occupational health management [28]. This is exacerbated by the ongoing stigmatization of mental health [29, 30].
3. Facilitation factors to the implementation of (general) occupational prevention measures in the inner setting include awareness of (long term) health risks, high commitment among employers and openness among staff, good communication strategies, integration in the organizational policy and high trust and autonomy of the employees [24]. A handful of previous studies in the setting of MSE have shown that the awareness of and knowledge about different forms of occupational stress prevention is low on average, especially when it comes to structural interventions [29, 30]. MSE have also been reported to consider the estimated benefit of stress prevention (specifically PRA) as too low [31].
4. Furthermore, in the individual domain, conducting a PRA involves some diagnostic skills from the employers or managers for the obtainment and interpretation of the questionnaire results, which might be a barrier for small enterprises (outside the health sector) without the necessary expertise [24]. Earlier qualitative studies have also stressed that owners of MSE regard prevention as a personal responsibility of the employee [32].
5. Finally, a few studies on barriers in the implementation process of general prevention programs (e.g. promoting exercise) show rather low participation rates (e.g. 47% in [18]).

Previous research provides a number of possible barriers to occupational stress prevention as well as factors that may cause it to fail. Nevertheless, to the best of our knowledge, there are no previous implementation studies with a comprehensive process evaluation of a complete implementation of occupational stress prevention in MSE.

### Web-based solutions for occupational stress prevention in MSE

Given the reoccurring issue of constrained resources and expertise in small enterprises, web-based interventions could offer a possible alternative for occupational stress prevention in MSE, as they are associated with flexible use and low costs. The effectiveness of web-based interventions has been shown in several studies:

Research in a large Dutch healthcare setting has shown that a web-based PRA could be effective for stress prevention at the organizational level [33]. Recently, more web-based PRA tools for structural interventions in smaller organizations have been developed [e.g. 34, 35], but not specifically for MSE. When developing an online training to facilitate the implementation of PRA, researchers from Germany and the Netherlands observed great interest from (representatives of) SME (42% of all participants) and participants reported a significant simplification of the process of PRA after the training [36].

For occupational stress prevention at the individual level, web-based SMT provides a way in which employees can train stress coping at anytime and anywhere, without disclosure to their employers or anyone else [37]. Meta-analytic evidence is still heterogeneous but shows that targeted web-based SMTs are effective in reducing stress and promoting well-being [38, 39]. Web-based SMTs including additional guidance from e-coaches show slightly higher effects than unguided (self-help) interventions [40]. Moreover, there have been first studies that indicate positive results in terms of cost-effectiveness [39, 41, 42].

However, there is a lack of implementation research for web-based SMT in the setting of MSE and it is still unknown if such systems can promote the implementation of preventive measures in small companies.

### Aim and research questions

The aim of the present study is to evaluate the implementation process of an integrated online platform for occupational stress prevention (combining established structural and behavioral preventive measures; named “System P”) in MSE. By addressing some of the barriers from previous studies, we hope to facilitate the uptake and use (implementation) of common stress prevention measures in MSE.

In the context of the present study, we explore different implementation outcomes according to Peters et al. [21] and use the CFRI framework [22] in order to evaluate specific determinants of the implementation in MSE from a quantitative and qualitative perspective. Besides important indicators of the intervention use (e.g., penetration, fidelity), we were especially interested in the implementation outcomes at the early stage of implementation (e.g., adoption, reach, acceptability and feasibility) to identify additional barriers and facilitators for the uptake and use of web-based stress prevention in this specific (undersupplied) setting.

The study is guided by the following research questions:

1. How can MSE be reached to implement web-based interventions for occupational stress prevention?
2. What kind of MSE decided to adopt and implement System P?
3. How did participating managers and employees of MSE use the web-based system (over a course of 6 months)?
4. How did MSE representatives and external stakeholders perceive the usefulness and fit of System P for the MSE setting?

The research questions will be answered in two parts, with a quantitative study focusing on questions 1), 2) and 3) and a qualitative study addressing question 4). The results will be reflected in a joint discussion at the end of this article.

## Part 1 – quantitative study

### Methods

#### Study design

**Fig. 1:**
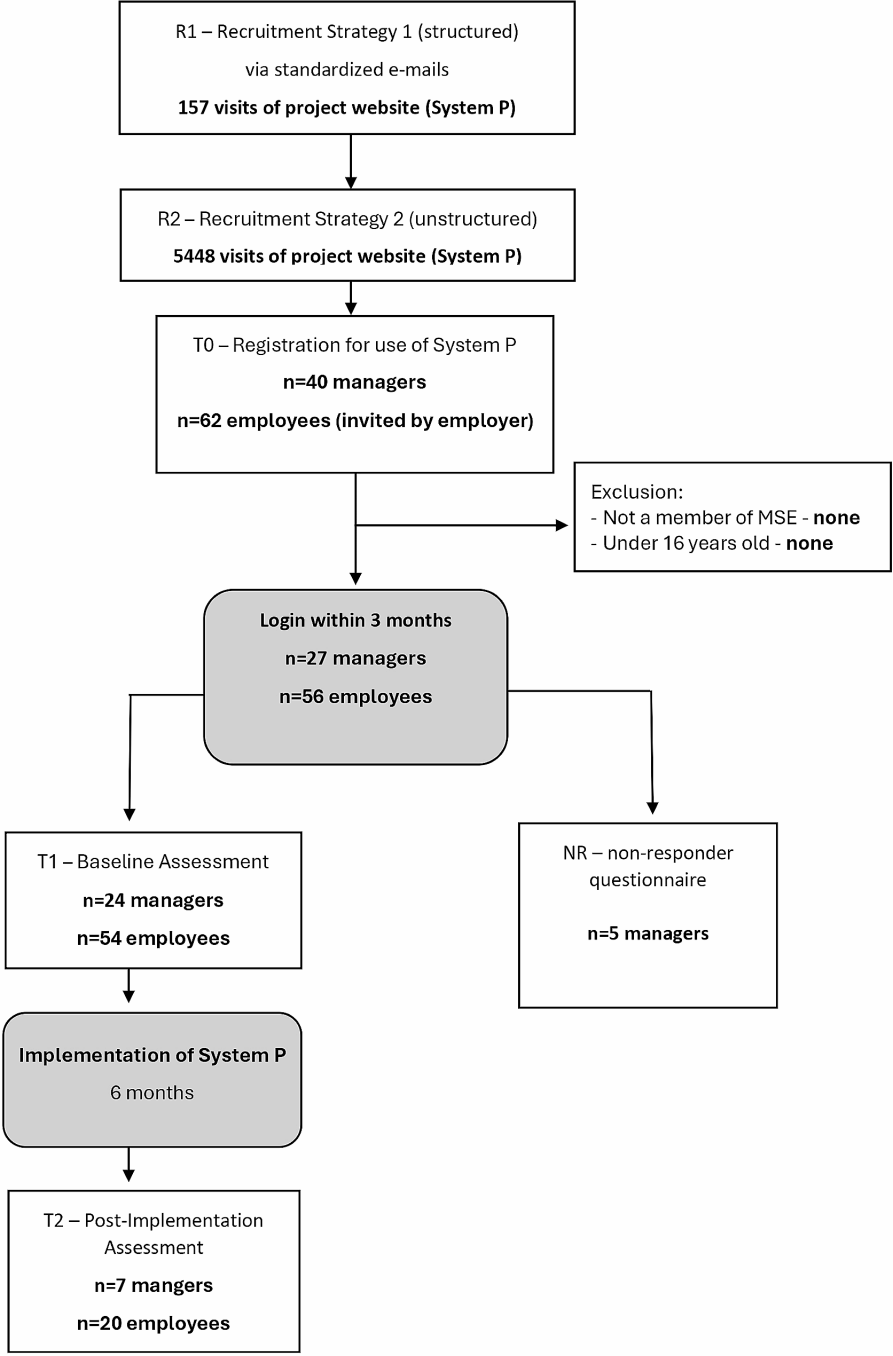
Study flow

This implementation study was conducted according to a previously published study protocol [43] and was approved by the ethics commission of the Medical Faculty of the Heinrich-Heine University Düsseldorf (reference No: 2021−1588). The implementation study originally followed a hybrid approach to implementation research [44] to evaluate both the implementation process and effectiveness of the web-based interventions in MSE at the same time. No adjustments were made to System P during the implementation study. However, due to a low number of registrations, the effectiveness of the interventions could not be analyzed appropriately. Therefore, this article focuses on the implementation outcomes from a quantitative and qualitative perspective. Part I of the study has a pre-post design with two measurements (T1 – baseline, T2 – after 6 months). Self-assessment questionnaires were administered directly via the intervention platform System P at baseline (after registration) and T2. During registration in System P, the potential participants received written study information. Before each questionnaire, the participants had to agree to a written declaration of consent. Figure 1 summarizes all relevant steps of this study part.

#### Intervention: integrated platform system P

For the purpose of this study, we combined existing web-based interventions for occupational stress prevention on structural and behavioral levels (PRA and SMT), implemented them into an integrated online platform, called “System P” and adapted the platform to the specific needs of MSE. For example, we developed features that allow MSE to independently carry out a PRA from anywhere without external help by use of a simplified approach with online questionnaires and automatized reports of the results.

More specifically, the web-based PRA intervention provides a simplified tool to follow a complete PRA cycle as recommended in the German guidelines for structural stress prevention [GDA; 45]. The original seven steps of the PRA cycle were summarized into three steps for managers to actively implement: (1) Preparation, (2) Analysis and Actions and (3) Evaluation, with some other measures (e.g., documentation) automated within the process. In step 1 of the PRA component, employers can generate customized questionnaires for their MSE to assess possible psychosocial risks (per division). PRA within System P provides a pool of 55 questions from validated questionnaires about the main psychosocial stressors at work (e.g., organization, workload, social support, physical environment and boundaries) [46, 47], which are expected to take between 30 and 60 min for individual employees to complete, depending on the number of chosen questions. The standard pre-selected questionnaire is a short version with nine questions on the most important stressors according to the GDA guidelines, which could be filled out in less than 10 min.

The web-based SMT is based on the established online training “GET.ON Stress” [48, 49]. In total, the SMT consists of seven sessions, which can be completed at your own pace. Yet, it is recommended to complete them on a weekly schedule [48, 49]. Each session is designed to last about 45 min, but can be interrupted at any time if necessary and continued at another time. The SMT includes two main strategies of stress coping: problem-solving [50] and emotion regulation [51]. GET.ON Stress has been shown to be effective in reducing stress and depressive symptoms in employees with elevated levels of perceived stress with intensive [48] and minimal guidance [52] as well as without any guidance [53]. It has also been shown to be effective as universal prevention [54] and for employees experiencing adverse working conditions [55]. Moreover, this intervention has been proved to be cost-effective compared to waiting controls [41]. Before integration into System P, the training was adapted to the MSE setting by introducing employees and managers of MSE as examples in the exercises [43].

System P further contains other non-intervention components that were designed to address common barriers to implementation. To overcome a potential lack of knowledge [25], the platform provides educational information about occupational stress prevention and its legal frameworks as well as mechanisms of work stress and health through a web-based “stress lexicon” (for a detailed description see appendix: 01 System P description). Although System P is available to registered users only, an overview of its most important benefits and three self-tests (checklist for organizational interventions, personal stress level and a quiz to test knowledge about stress) are provided on the public landing page in order to emphasize the usefulness of occupational stress prevention. The platform includes short video and audio instructions to increase accessibility as well as application examples from different industrial sectors. Typical obstacles in the implementation process with recommended solutions (e.g., communication towards employees) are listed in a section with frequently asked questions (FAQ) (see appendix: 02 FAQ). The manager version of System P also provides access to a moderated forum where employers can share advice and experiences [27] (see appendix: 03 Forum). The project team organized regular one-hour introductory webinars to explain all components of System P, which could be attended live or viewed afterwards.

The full development of System P has been described in the study protocol [43].

#### Recruitment

A two-stage recruitment strategy was developed through an extensive media and literature analysis to address and activate MSE managers to register their enterprise and subsequently invite their employees to participate. Recruitment strategy one (R1) included a structured approach and addressed MSE managers via e-mails sent out by external recruitment partners. Recruitment partners were institutions and networks who support occupational health and safety activities and are already known by MSE (e.g., accident insurance institutions, health insurance companies, company physicians and local networks). The standardized e-mail contained an invitation to register on System P, information about System P and an individualized link to the project website (see appendix: 04 Translation of standardized e-mail). On the project website, MSE managers received information about System P, including a short introduction video, a visual summary of the advantages of occupational stress prevention, self-administered tests and checklists, best practice scenarios and the opportunity to participate in an introduction event (or watch a recording of it). Interested MSE managers could register for System P if they fulfilled the inclusion criteria. Participants had to be either a manager of an MSE (enterprise with less than 50 employees – full-time equivalent) or an employee of an MSE invited by their manager. Participants were excluded from the study if they were under 16 years old. There were no further exclusion criteria.

Recruitment strategy two (R2) involved an unstructured approach in order to recruit additional participants for the study. The communication channels in R2 involved, among others, distributing information about System P via printed flyers, face-to-face seminars for MSE conducted by the recruitment partners, online distribution via newsletters and online posts on websites of recruitment partners, extensive articles in organizational health and safety journals or magazines, as well as presentations during events of recruitment partners and personal presentations for interested MSE. In addition, R2 involved an extensive social media campaign and search engine advertising (see appendix: 05 Example image social-media post).

Dealing with non-responders during recruitment: Participants who registered but did not fill out the baseline questionnaire after 3 months (non-responders = NR) were asked about their reasons for not using System P via a short e-mail survey with open questions. The NR feedback could be sent openly via a direct response to the e-mail or via a link to a short structured anonymous non-responder questionnaire.

#### Measurements and procedure

Socio-demographic variables of all respondents were collected at baseline measurement (age, gender, family status, first language, education, occupational position, working hours, income, ). Managers were additionally asked to provide information about the enterprise (number of employees, history of health program use in the organization, location (region) and industrial sector).

To systematically evaluate the success of the implementation of System P in the context of MSE, we collected data on the following outcomes as defined by Proctor et al. [21]: adoption/reach, appropriateness, feasibility, fidelity/dose, penetration, acceptability and costs. These outcomes specifically relate to the quality of the implementation (process) and help to map the (potential) real-world impact (or failure) of an intervention in a specific setting [21]. Each outcome has a theoretical basis and the implementation stages at which the outcomes are most salient vary. Implementation outcomes can be linked to different determinants in the CFIR framework [22], which helps to explain the necessary preconditions as well as potential barriers for successful implementation. Sustainability, another outcome defined by Proctor, was not included because of a relatively short follow-up period. Table 1 provides an overview of the operationalization of the main quantitative outcomes (see study protocol for further details).

**Table 1:** Summary of quantitative measures of implementation outcomes for System P

Implementation outcome	Research question	Definition	Operationalization	Time of data collection	Relevant domains within CFIR
Adoption	1	Initial decision to implement an intervention	Number of registered MSE / number of invited MSE at R1	*R1 and T0*	*innovation (intervention) characteristics* *outer setting*
Reach	2	Reach of intervention within population	Comparison of socio-demographic data of sample and population	*R1 and T1*	*innovation (intervention) characteristics* *inner setting* *individuals*
Penetration	2	Distribution of intervention within organization	Number of employees who filled out T1 / total number of employees as reported at T1 (target participation rate of 50% or more)	*T1*	*inner setting* *individuals*
Fidelity	3	Adherence to the implementation protocol	Reported time spent in System P vs. actual time spent in System P Order in which components are used	*Between T1 and T2*	*implementation process*
Dose	3	Amount of intervention received by participants	Number of logins Number of completed steps in PRA (minimum 2) and lessons in SMT (minimum 5)	*Between T1 and T2*	*implementation process*
Costs	3	Perceived costs of intervention	Perceived time investment: reported time spent in System P vs. actual time spent in System P	*T2*	*inner setting* *implementation process*
Acceptability	4	Perceived usefulness of intervention	Readiness for change Average ratings of intervention and user experience (average 3 or higher)	*T1* *T2*	*implementation process* *innovation (intervention) characteristics* *individuals*

#### Operationalization of implementation outcomes

Adoption: Adoption refers to the initial action taken by an organization interested in implementation (in our case: registering for the use of System P) [21]. Adoption rate was calculated by dividing the number of registered MSE at T0 by the number of MSE invited via e-mail during recruitment strategy one (R1). We additionally calculated the number of website visits per individualized link of our recruitment partners to see whether general interest varied by network/industry.

Reach: Reach was analyzed by comparing socio-demographic characteristics of MSE at T1 with data from the German population, partly gathered as aggregated data from our recruitment partners and partly from earlier representative studies. MSE were compared with regard to company size, industry and prior experience with occupational (stress) prevention.

Penetration: Penetration of an intervention refers to the reach within the participating organization to see if all employees can potentially benefit from it. It was analyzed by dividing the number of employees who filled in a baseline questionnaire by the number of actual employees in the MSE (as reported by the employer at T1).

Fidelity & Dose: To evaluate the adherence to the intended implementation process, we collected usage data directly via System P. Important indicators of dose were the number of logins, the number of steps completed in the web-based tool for PRA (minimum two of three) and the number of completed sessions in the web-based training (minimum five of seven).

Acceptability: In the quantitative measurements at T1 and T2, general acceptability of stress prevention interventions was assessed with three items on “Readiness for Change” adapted from earlier process evaluations for stress prevention [56] (scores 1 “do not agree at all” to 5 “completely agree”). Both managers and employees were asked whether they agreed that occupational stress prevention is valuable, positive for the organization and necessary. For a better classification, mean scores were grouped into three categories: high (4.0–5.0), moderate (2.6–3.9) and low (1.0-2.5) readiness for change. At T2, users were also asked to give a rating of one to five star to evaluate the usefulness of each intervention component of the system. Another indicator of acceptability was the user experience at T2. User experience was assessed with the safety subscale of the System Usability Scale [57] and the Questionnaire for Modular Evaluation based on the Components model of User Experience (meCUE) [58], which includes the aspects of visual aesthetics, usability and usefulness.

Costs: Although the use of the System P was free of charge, companies still required (human) resources to implement the web-based interventions. Therefore, usage times served as an estimate for the indirect costs for MSE and the providers. Hours spent on the web-based platform were assessed at T2 and login times were monitored for managers, employees and e-coaches over the course of the 6 months.

#### Additional measures

The following measures were additionally included in the questionnaires at T1 and T2 to account for pre-existing differences between MSE:

General conditions regarding stress prevention (inner setting) were measured with a German adaptation of the short version of the Psychosocial Safety Climate Questionnaire (PSC-4; [59–61].

We also assessed individual levels of mental health before and after the implementation (T1 and T2). The primary indicator were depressive symptoms measured by the short version of the Patient Health Questionnaire (PHQ-8) [62]. The eight items of the PHQ-8 describe different depressive symptoms and ask how often they have occurred in the last two weeks (scores 0 “not at all” to 3 “almost every day”). Usually, the cut-off score for clinical relevance is ten [62].

Finally, the general attitude towards new technological systems was measured with four items of the Affinity for Technology Interaction Short Scale (ATI-S; [63]) for managers and two items for employees. The ATI-S is a four-item scale with scores ranging from 1 “not at all true” to 6 “completely true”. The questionnaire asks whether the participants are generally open to new technologies and whether they want to try out new functions (in contrast to being satisfied with just basic functions). The employees were only asked about their openness towards the use of new technologies or new functions of a technology.

Further details on all measurements and target values for the implementation outcomes can be found in the original study protocol [43].

#### Analysis

Due to the high drop-out between T1 and T2, analysis of quantitative data is restricted to descriptive results. Multilevel analysis with sufficient power was not possible. The quantitative analyses were calculated using IBM SPSS 27. A response rate was analyzed for structured recruitment. Furthermore, frequencies for the use of System P were calculated as well as mean values and sum values. A detailed description of the evaluation process allows for a specific overview of the reached target group and the adoption and use of System P. To explore possible determinants associated with characteristics of the individual and the inner setting, we compared the descriptive values of the individuals and MSE who participated in the study with the general German working population, as well as benchmark and reference values from other studies (e.g. psychosocial safety climate scale).

### Results

#### Adoption

In R1, a standardized e-mail was sent to a pre-determined number of MSE (*n* = 5413) by recruitment partners. Knowing the exact number of contacted MSE allowed us to track their adoption behavior and calculate an adoption rate. In response to the standardized e-mails, the project website was visited 157 times within the time of analysis (i.e., between December 2021 and January 2022)^1^. On the project website, the short introduction video of system P was watched 57 times and seven visitors enrolled for the introduction event. In total, seven of these MSE registered for System P. Thus, addressing MSE managers via standardized e-mails and recruitment partners in R1 yielded an adoption rate of 0.13%.

**Fig. 2:**
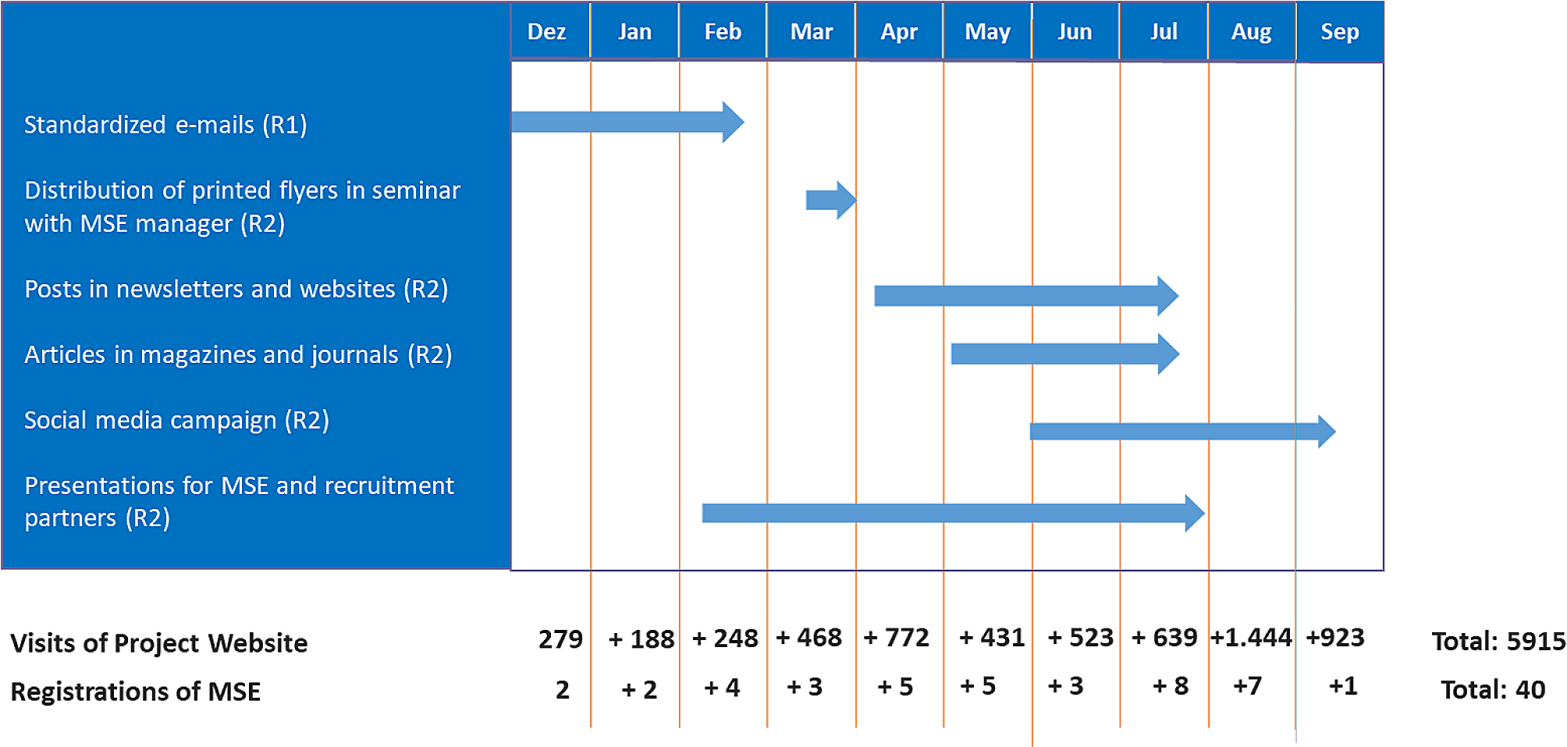
Timeline of used recruitment channels during structured and unstructured approach

Figure 2 provides an overview of the timeline of R1 and R2 as well as the resulting number of visits to the project website and subsequent registrations^2^.

In R2, additional communication channels were used to increase the number of MSE registering in System P. R2 started in February 2022 and was unstructured, as the number of MSE managers that could potentially be reached via the different communications channels was unknown. Hence, it was not possible to calculate an adoption rate for R2.

**Fig. 3:**
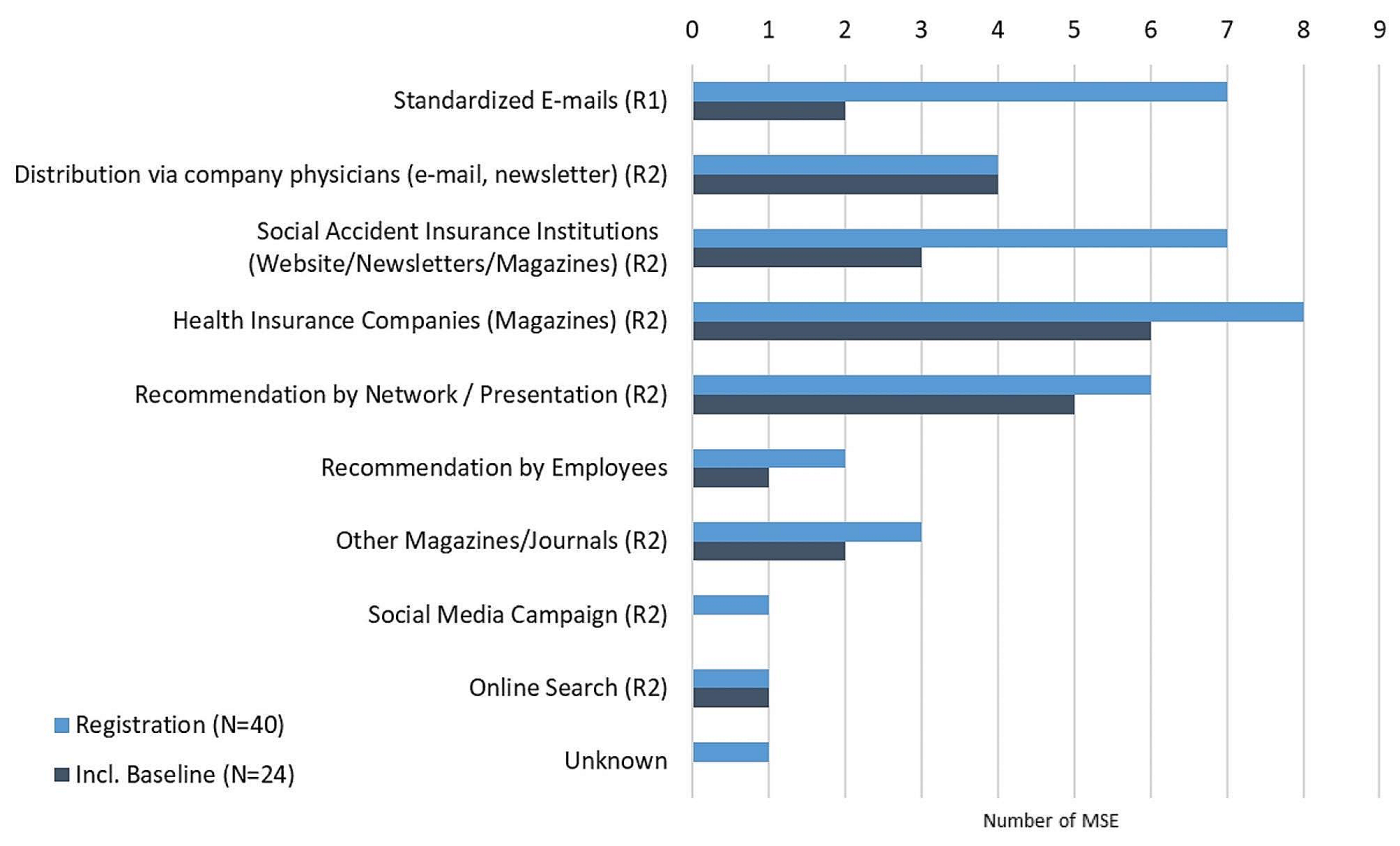
Numbers of MSE by communication channels via which they were contacted (own info)

In total, 40 MSE registered to use System P as a result of the two recruitment strategies. Figure 3 indicates how these 40 MSE learned about System P and further differentiates between MSE who only registered in System P and MSE who also filled in the baseline questionnaire.

During the registration period from December 2021 to September 2022, 102 participants from 40 MSE (40 managers and 62 employees) registered in System P. Out of the 40 MSE registrations, 15 managers and eight employees did not complete the enrollment process or never filled out the baseline questionnaire. These managers and employees were contacted again after three to six months and asked to provide short feedback on why they did not use System P. From a total of 19 NR contacted, five NR replied. Reasons given were: not enough time to use System P, other important operational issues with higher priority in the enterprise and (mis-)believing that the size of the enterprises was too small to implement the measures in System P.

#### Penetration

Twenty-four of the registered 40 managers (representing their MSE) completed the baseline questionnaire (60%). These 24 MSE reported having a total of 359 employees. However, only eight of the 24 MSE invited some of their employees to use System P. Out of the 91 invited employees, 62 registered and 54 completed the baseline questionnaire (response rate of 59.3%). The overall resulting penetration rate (participating employees / all employees of the participating MSE) was 15%, with only 3 MSE having more than 50% of their staff included in the web-based stress prevention program.

**Table 2:** Description of the study sample: managers and employees

		Managers (*n* = 24)	Employees (*n* = 54)
		mean (SD)	mean (SD)
Age	Years	45.46 (9.90)	36.43 (9.35)
		n (%)	n (%)
Gender	Female	13 (52.4)	38 (70.4)
Length of employment	> 10 years	9 (37.5)	1 (1.9)
	6–10 years	6 (25.0)	6 (11.1)
	3–5 years	5 (20.8)	12 (22.2)
	1–2 years	3 (2.5)	18 (33.3)
	< 1 year	1 (4.2)	17 (31.5)
Vocational qualification	PhD (promotion)	3 (12.5)	9 (16.7)
	University graduation	13 (54.1)	26 (48.1)
	Vocational training / technical apprenticeship	4 (16.7)	11 (20.4)
	Master craftsman, technician, vocational school	4 (16.7)	4 (7.4)
	No vocational qualification		4 (7.4)
		(*n* = 7) mean (SD)	(*n* = 24) mean (SD)
Gross personal wage	Euros per month (average)	4.807,14 (1.305,90)	4.051,54 (2.902,03)
PHQ-8		mean (SD)	
	Baseline (*n* = 73)	6.99 (5.31)	
	T2-Participants at baseline (*n* = 26)	6.81 (5.10)	
	Follow-up (T2) (*n* = 24)	6.60 (5.82)	

Note: Percentages are given with one decimal place, all other values with two decimal places

#### Reach

Of the participating companies 58.3% were small-sized enterprises with 10–49 employees, while the remaining 41.7% were micro-sized enterprises with one to nine employees. Regarding the industrial sectors, healthcare and social services were overrepresented with 7 MSE. Other indicated sectors were professional, scientific and technical services (6 MSE), other services (3 MSE) as well as construction and trade (2 MSE) and manufacturing/production of goods (2 MSE).

Compared with the general German working population, the individual participants in the study had higher educational and vocational qualifications and were slightly younger. Among managers, 58.3% and among employees, 77.8% had a high school diploma, compared to 34.5% in the general working population [64]. The proportion of women in the present study (managers 54.2% and employees 70.4%) fits to the general figures in healthcare and social services in Germany [65].

For detailed sample information see Table 2.

**Fig. 4:**
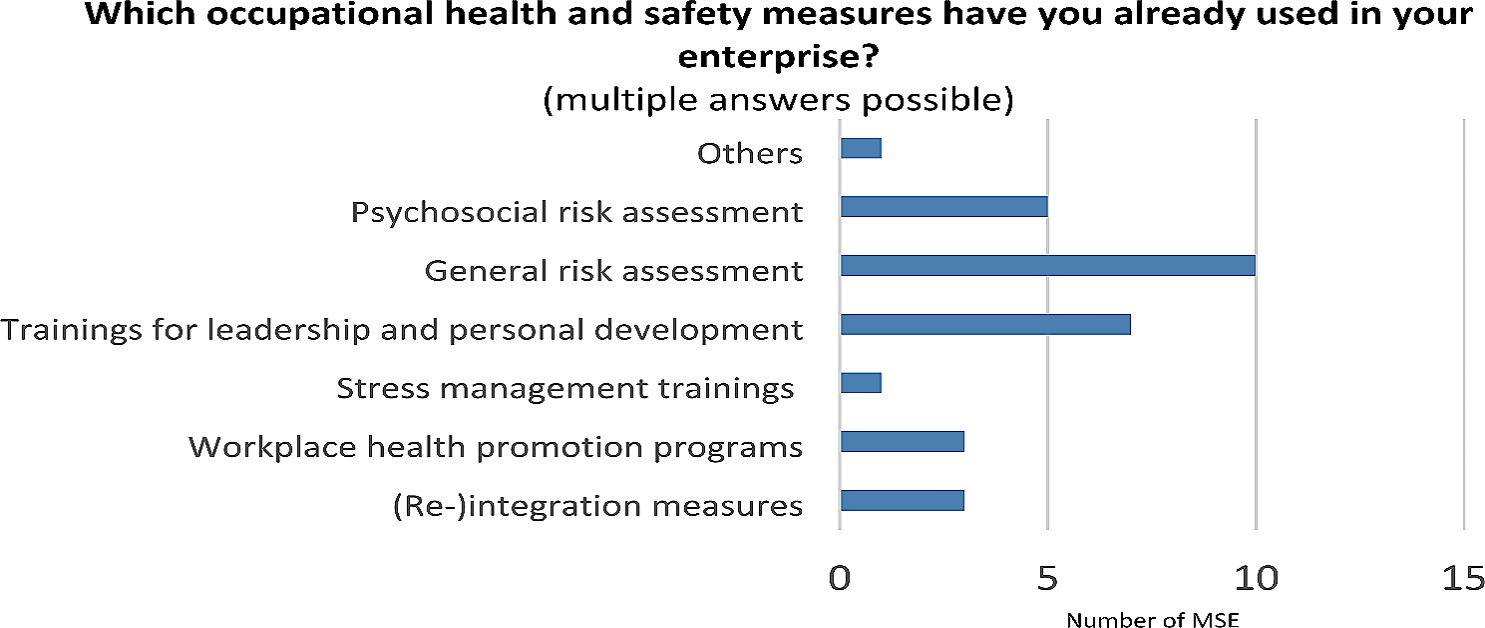
Previous experience with occupational prevention measures (*n* = 15)

It is important that, the majority of participating MSE reported having prior experience with occupational health measures and 62.5% had already implemented occupational stress prevention interventions (PRA or SMT) in the past (see Fig. 4).

**Fig. 5:**
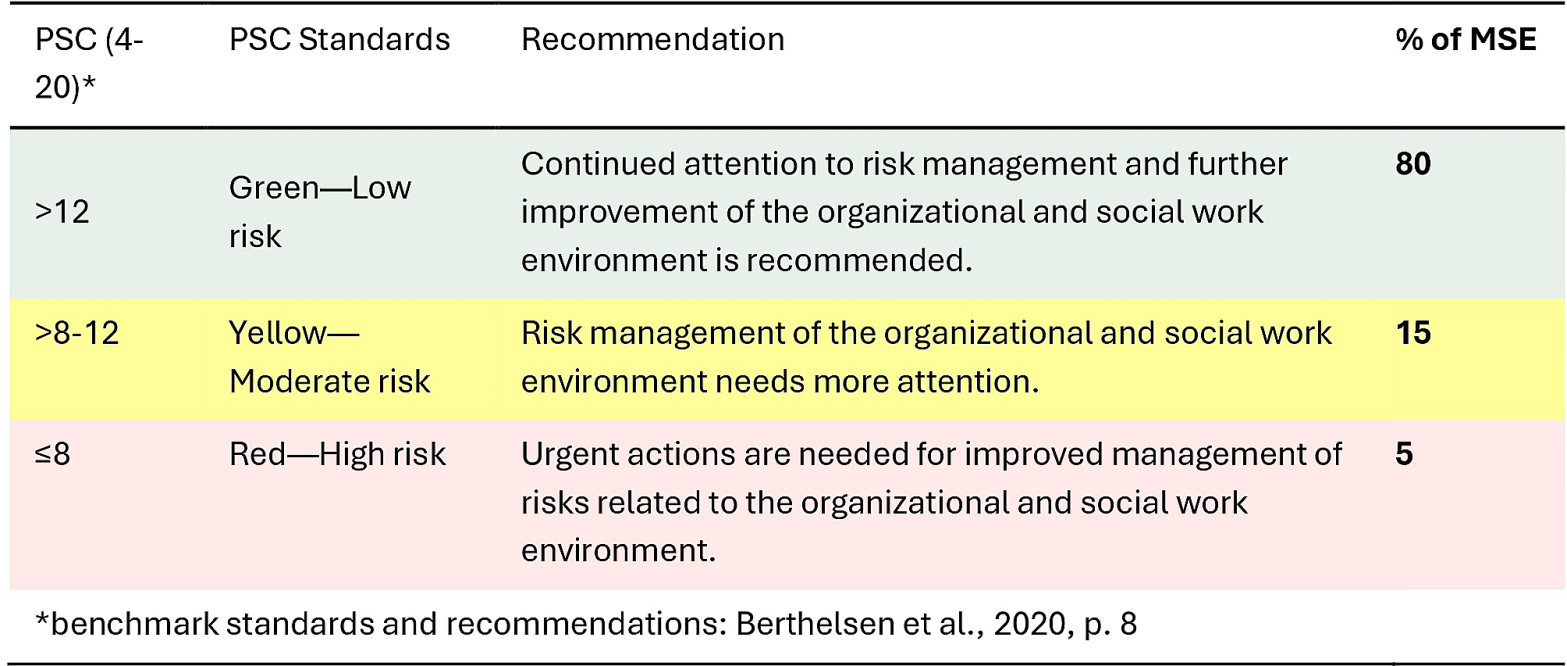
Benchmark standards and recommendations [59]

The analyses of the Psychosocial Safety Climate (PSC) revealed a generally good climate in the participating MSE according to the benchmark standards and recommendations of Berthelsen et al. [59] (see Fig. 5). At the MSE level, 80% had scale values indicating a good PSC (mean 15.20 and 3.35 SD, *n* = 20). An additional 15% of MSE had a moderate PSC for implementing psychosocial occupational health and safety measures and only one enterprise ranked below the cut-off value of eight [61].

The analysis of other characteristics revealed that the managers were mostly affiliated with technology (ATI-S: Managers: M = 3.85; 0.60 SD). Employees were also willing to try out new functions (74.1%) and were interested in new technical systems (70.4%).

#### Acceptability

On average, the readiness for change was high among managers (mean 4.36 (0.59 SD)) and employees (mean 4.13 (0.61 SD)) [56, 66]. When looking at the readiness for change categorically (high, moderate and low readiness for change), 86.4% (*n* = 19) of managers and 69.8% (*n* = 37) of employees showed a high readiness for change, believing in the value of stress prevention interventions. A moderate readiness for change was found among the remaining users.

**Fig. 6:**
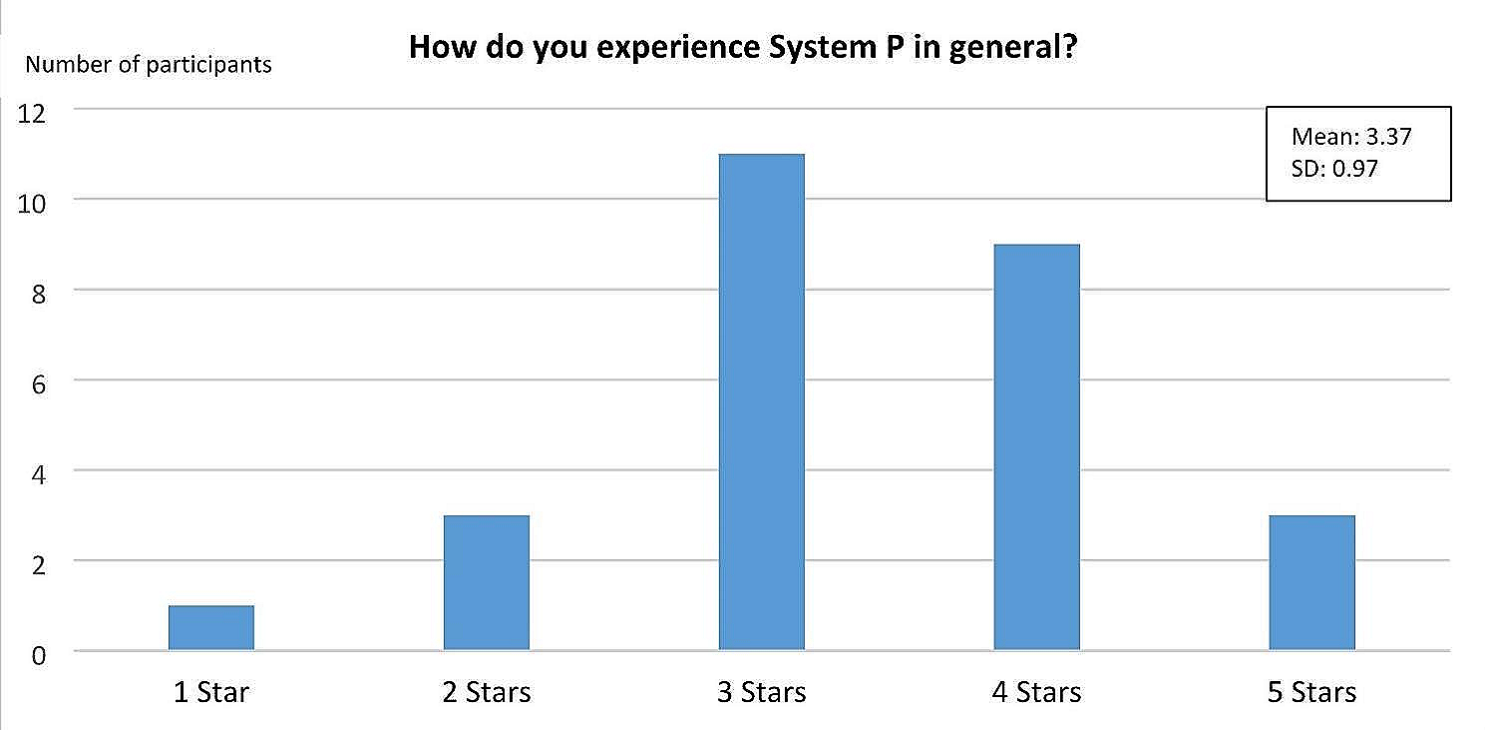
General experience with System P at T2 (*n* = 27)

General acceptability of System P at T2 was measured by a simple star-rating (1 = lowest and 5 = highest acceptability) or higher. More than two-thirds of the participants rated their experience with the system with three or more stars (see Fig. 6). Overall, the user experience in T2 (*n* = 10) regarding the interaction with System P was satisfactory (mean = 2.83 (SD 0.88)). The usability aspect was rated highest by the participants (*n* = 14) (M = 3.62; SD 0.91), which showed that System P was commonly perceived as easy to use and that participants were able to quickly learn how to operate. Likewise, the design of System P (stylish, creative, attractive) was perceived as satisfactory by the users (*n* = 12) (M = 2.86; SD 1.15). Regarding the aspect of usefulness (*n* = 10), i.e., the extent to which System P is useful in achieving the user’s goals in the area of stress prevention, System P was rated as partially satisfying (M = 2.43; SD 1.04).

#### Fidelity and dose (usage data)

The minimum number of completed steps for the web-based tool for PRA was set at two out of three. No MSE finished step two of the web-based PRA, but one MSE started to implement a measure to improve working conditions. In total, eleven MSE initiated a questionnaire-based assessment of psychosocial risks in the first step of the web-based PRA and invited 86 employees. The surveys of the web-based PRA were answered by 31 employees. Only two MSE looked at the results. On average, 31 questions were included in the survey, mainly questions related to the nine key factors of the PRA. Out of the eleven MSE that had sent out a survey, eight MSE used the standardized short version of the questionnaire (44 items), two MSE only used the questionnaire concerning key factors (9 items) related to the German guidelines for PRA and one MSE used the full questionnaire (64 items). Two MSE additionally developed their own questions. Beyond the key factors, managers were particularly interested in topics such as professional development, team, responsibility and accountability. The results of the key factors in the PRA step one (*n* = 31) showed that the employees were not exposed to any serious work-related stress, except for the area of work organization, where 33.3% of the employees (*n* = 10) stated that they often or always have to work under time pressure. However, most employees only worked overtime occasionally (46.7%; *n* = 14). At the end of the observation period, no MSE in System P had implemented a targeted action to counteract risks (Step 2) or carried out an assessment of the targeted measures (Step 3).

The web-based SMT was initiated by 25 users. Nine participants completed the minimum number of training sessions (five out of seven) and thus achieved the implementation goal. Of these, eight participants, including two managers and six employees, completed the entire training. However, a more detailed analysis of the usage data of the web-based SMT shows that only three people engaged in the exercises of the individual sessions in the SMT as intended.

#### Costs

To evaluate indirect costs, the managers in T2 were asked how much time the processing of the web-based PRA had taken. In addition, the corresponding usage times for the first step (the employee survey) of the PRA were recorded for the managers. In T2, the managers (*n* = 4) stated that they had spent an average of two and a half hours with the PRA. According to the System P usage data (*n* = 13), the processing of the first step of the PRA took an average of 17.1 min (with a maximum of two hours). The same was applied for SMT use. In T2, participants (*n* = 10) stated that they had spent an average of one hour with the SMT. According to System P usage data (*n* = 23), the average time spent in the SMT was 13.4 min. Each training session is scheduled to last about 45 min (seven training sessions in total).

In total, participants logged in 302 times after the baseline assessment. Until the end of the observation period, the most active managers logged into the system 47 times. The average number of logins was 6.3 per manager. Employees logged into System P notably less often than managers. The most active employee logged in nine times within the observation period. The average number of logins was 2.8 per employee.

To enable participants to use System P as efficiently as possible, all users had the possibility to ask questions about the content or request help with technical difficulties via a support service when using the system. The support could be contacted by e-mail, or a return call could be requested. The support received a total of 52 requests from December 2021 to March 2023. There were only a few requests regarding content, e.g., inviting employees to System P. Most support requests were technical issues, e.g., regarding registration or access to the PRA results. None of the participants made use of the optional individual e-coaching.

## Part 2 – qualitative study

### Study design

The qualitative part of the study consists of semi-structured interviews conducted with managers of MSE and intercorporate stakeholders, e.g., occupational physicians or safety supervisors. Three researchers were responsible for the conception of the study and the analysis, and three researchers conducted the interviews. The research team is experienced in occupational health research and qualitative research methods. Due to the low adoption rate for part I, the interviews took place in parallel with an independent sample, deviating from the study protocol [43]. The sample included (a) managers of MSE who were not drawn from the registered users of System P and (b) intercorporate stakeholders who were experts on occupational prevention in the context of MSE. The research team aimed to identify barriers to adoption for potential users and experts. A corresponding interview guide (see appendix: 06 Interview questions managers and stakeholders) was pilot-tested.Recruitment procedure.

Participants for the interviews were recruited at the same time as participants for the quantitative study (R2). To facilitate the recruitment process of the qualitative study, a convenience and snowball sampling approach was selected, and participants were mainly recruited through personal contacts within the research environment and via the recruitment partners.

### Participants

A total of six managers of MSE and six intercorporate stakeholders took part in the interviews between February 2022 and August 2022.

Four of the participating managers were female and had an average age of 49 years (11.21 SD). In terms of business size and sector, four managers led a micro-sized and two a small-sized enterprise in the following sectors: craft (*n* = 3), healthcare (*n* = 2) and tourism (*n* = 1). Of the participating intercorporate stakeholders two were female and on average 44 years (9.88 SD) old. They were occupational physicians (*n* = 2), (digital) coordinators for occupational health management (*n* = 2), a safety supervisor (*n* = 1) and a funding supporter for start ups (*n* = 1).

### Measurements and procedure

To gain further information about potential barriers to the implementation of System P, we collected additional data on the implementation outcomes acceptability, appropriateness and feasibility [21] (see Table 3).

Acceptability: Based on three short video sequences introducing the web-based platform, managers and intercorporate stakeholders were asked about the expected advantages and disadvantages of implementing System P and whether managers intended to use System P.

Appropriateness & Feasibility: During the interviews, managers of MSE and stakeholders were asked about the perceived fit of System P for the setting of MSE in general and for their own MSE. Additionally, participants were asked which components they liked and disliked and whether the instructions for the use of the system were clear. The semi-structured interviews also included questions about potential barriers to the use of the platform.

The interviews were conducted via a web conferencing software with audio recording. Each participant received written study information before the interview and gave written consent to participate. The duration of the interviews was 27 to 50 min for the managers, and 24 to 68 min for the intercorporate stakeholders. While conducting the interviews, the research team regularly discussed data saturation and concluded in the end that 12 participants was a sufficiently large number to reach it.

**Table 3:** Summary of qualitative measures of implementation outcomes for System P

Implementation outcome	Research question	Definition	Operationalization	Relevant domains within CFIR
Acceptability	4	Perceived usefulness of intervention	Open questions about impression of intervention components	*Innovation (Intervention Characteristics)*, *Individuals*
Appropriateness	4	Perceived fit between intervention and setting	Open questions about intervention components after video demonstration	*Innovation (Intervention Characteristics), Inner Setting*
Feasibility	4	Perceived (and actual) barriers to use	Questions about potential barriers to implementation	*Innovation (Intervention Characteristics), Inner Setting* *(Outer Setting)*

### Data analysis

Qualitative data were analyzed by transcribing the recordings and applying qualitative content analysis as described by Kuckartz [67]. The software MAXQDA 2020 was used. In this approach, a dynamic categorization system that is replicable and valid was developed by three researchers. With regard to the coding of the data, a mixed deductive-inductive approach was used. The deductive approach comprises the development of main categories based on the literature and the research questions, whereas the inductive approach includes the development of subcategories from the interview data. Three researchers conducted these two coding approaches in combination with regular intensive discussion aiming for consistent coding. Additionally, three researchers reviewed the category system and coding for plausibility, consistency and interpretability. The results of the content analysis were summarized according to indicators of a successful implementation process, specifically addressing acceptability, appropriateness and feasibility [21] and the five domains of determinants according to the CFIR: innovation, outer setting, inner setting, individuals and the implementation process (see Table 3).

## Results

In an overall assessment, System P was predominantly evaluated to be acceptable and appropriate from the perspective of MSE managers and intercorporate stakeholders, especially in relation to the operating conditions of MSE. With regard to the feasibility of the intervention, results are mixed in terms of intervention format, adaptability, complexity and indirect costs (e.g., time investment) (see Table 4 for a summary).

### Acceptability

Results predominantly showed that System P was acceptable and satisfactory from the perspective of managers as well as intercorporate stakeholders. Positive beliefs about the intervention prevail, the perception of advantages was much stronger than the perception of disadvantages and the entrepreneurs mainly favoured the use of the intervention.

As an indicator for acceptability, we first analyzed the knowledge and (perceived) beliefs about the intervention [22]. Regarding the knowledge about the intervention, the functions of the system and its components were understood very well by the participants. The participants expressed mainly positive and a few negative viewpoints about the intervention: The managers described both PRA and SMT as an opportunity to learn and reflect about problems at the workplace. While most of them perceived the additional components (e.g., stress lexicon and forum) of System P as useful, others stated that too many features could discourage the use of the system. Intercorporate stakeholders emphasized that System P meets the needs for tailored occupational stress prevention in MSE and described the platform as a “bundling of good offers” (IS4) and “help for self-help” (IS1). Some stakeholders believed that convincing MSE managers about the usefulness of the system could be difficult and that the implementation of the structural intervention modules requires expert knowledge so that it cannot necessarily be performed by MSE without third-party support. Apart from that, the managers considered a support of intercorporate stakeholders in the introduction of the system as beneficial for employee acceptability.

Furthermore, the participants approved the intervention source. They perceived the externally and independently developed platform as positive for employees´ acceptance. Additionally, the intercorporate stakeholders evaluated the evidence strength and quality of System P as positive due to the scientific findings behind the intervention modules.

We furthermore investigated the perception of advantages and disadvantages of implementing the intervention [22] as a key indicator of acceptability. Expected benefits were, among others, the removal of taboos concerning work stress, improvement of working conditions, fulfilment of legal requirements, increased team bonding and employee satisfaction, motivation, increased productivity, improved employee health and well-being and prevention of mental illnesses, absenteeism and employee fluctuation. Managers described a variety of possible gains for themselves and their companies (e.g., showing interest in employees’ health and improving their own stress management). Expected disadvantages included increased workload and time investment at both levels as well as psychological pressure for employees to take part.

The interviews revealed a general tension caused by societal changes and the managers’ aspiration to lead a sustainable, successful company. Reasons for the use of the system were, among others, a perceived need for and individual interest in occupational stress prevention as well as signalled interest in employee well-being and in increasing job satisfaction. Arguments against usage were data protection concerns and time intensity of the intervention. In sum, the results predominantly show a tendency of the managers towards accepting and implementing System P.

### Appropriateness

To investigate the appropriateness (the perceived fit of an intervention for a specific setting) of occupational stress prevention with System P in MSE, we analyzed relevant CFIR domains such as: the inner setting of the company, characteristics of the managers, culture/implementation climate and perceived costs.

Generally, some participants indicated that the web-based intervention is fitting for small businesses, while others highlighted that the interventions only suit larger enterprises and that no “bureaucratic monster” (MSE5) is needed. The intercorporate stakeholders tended to consider System P as suitable for small (not micro-sized) enterprises in general. Facilitating conditions for a good fit were the prevailing open climate of change and the conviction of the managers, which came along with the willingness to concede time for the use of the system, as well as a generally available willingness of the employees for the implementation of the intervention. Furthermore, the participants perceived a fit for companies with a high degree of digitalization. In this context, some stakeholders affirmed that MSE in general have the necessary digital competencies to use the system, while others suggested that not every enterprise has them. System P was seen as more appropriate for MSE with a preference for structured work and compliance with legal requirements and for MSE with an open team culture and prior experience in the use of formal and/or digital interventions. Some intercorporate stakeholders indicated that occupational stress prevention is not seen as a priority in many MSE, so that raising awareness for occupational stress prevention is difficult. In contrast, others emphasized the increasing awareness for work stress, the willingness to use prevention offers like System P and good communication due to the size of MSE. The assessment of perceived indirect costs in terms of time investment was mixed, both appropriate and inappropriate.

### Feasibility

Participants named a few additional barriers to a successful implementation in the setting(s). While some participants appreciated the digital format, which enables flexibility in use (i.e., from home or after working hours), some preferred a non-digital approach. The intercorporate stakeholders emphasized that accompanying face-to-face support in addition to the digital approach would improve feasibility.

Findings with regard to the adaptability of the system were mixed. Some of the participants valued that the PRA can be easily adapted to the needs of the enterprise (including question pool, different assessment modes, differentiation of departments) and that the modules of System P were optional to use (e.g., FAQ, forum). In contrast, some other participants assessed the high number of features as potentially overwhelming. The complexity of the intervention is a further important factor for feasibility. With respect to the design complexity, participants valued the structure of the system, which provides a good overview of the modules and is easy and intuitive to use. On the other hand, the text load within the system was mentioned as a barrier. Regarding the pool of interventions provided by the system, some participants perceived the level of complexity as adequate, while others raised concerns that it was too complex and described the interventions as challenging to put into practice (i.e., difficult to implement measures after Step 1 of PRA). Some participants of both groups mentioned that managers need to be able to communicate the benefits of stress prevention to their employees and stated that they might lack the specific knowledge about occupational stress prevention to be able to use System P appropriately without a supporting third party.

Barriers mentioned by the managers were the fact that users need to be able and willing to invest time in using System P. This means that managers need to provide their employees with the respective time. There was a general disagreement with regard to the feasibility of the time investment/indirect costs (partly related to an uncertainty about the actual time necessary for a successful implementation). The stakeholders perceived the communication regarding the implementation process as challenging and even more time-consuming than the actual use of the system.

**Table 4:** Summary of qualitative results for System P

Implementation outcome	CFIR domain	Determinants within domain	Evaluation by managers	Evaluation by stakeholders
Acceptability	*Intervention Characteristics*	Knowledge and beliefs about the intervention	+	+
		Intervention Source	+	+
	*Individuals*	perception of advantages	+/-	x
Appropriateness	*Intervention Characteristics*	Digital format	+	+ -
		Perceived (indirect) costs	-	-
	*Inner Setting*	Company size (suitable for MSE)	+/-	+/-
		Change Climate	+	+
Feasibility	*Intervention Characteristics*	Digital format	+/-	+/-
		Adaptability	+/-	+/-
		Design	+	+
		Complexity of content	+/-	+/-
	*Inner Setting*	Communication efforts	-	-
	*Outer Setting*	Available Resources	-	x

Note: - = negative; + = positive, +/- = mixed, x = no information

## Discussion

The aim of the present study was to comprehensively evaluate the implementation of web-based interventions for stress prevention in MSE. For this purpose, a combined web-based platform (including PRA and SMT) was developed considering the specific needs of MSE and designed to enable location- and time-independent stress prevention without external help. We observed the implementation process in 40 MSE over the course of 6 months and evaluated the success of System P according to the implementation outcomes by Proctor et al. [21]. We also analyzed possible determinants according to the CFIR [22].

In the past, stress prevention has hardly been implemented in MSE [17]. The reasons for these low implementation rates are manifold, with the main barriers being the perceived complexity of prevention measures for implementation in MSE [24] and a lack of resources [24, 26]. System P addressed these barriers (e.g., through a simplified PRA and location- and time-independent access) in order to increase the likelihood of an adoption of the interventions. The results of the study show that System P partly fell short of the expectations regarding the outcomes of the implementation (process) within the observation period. System P reached only a small part of the target group, which was already sensitized to stress prevention. However, even in this informed and engaged group, usage was low in terms of fidelity and penetration, although System P was accepted by users and received good ratings for usability and appropriateness.

With regard to the first two research questions (How to reach MSE and which MSE decide to implement the system? ), it becomes clear that despite extensive recruitment strategies, only a small part of the target group could be reached. These were in particular MSE who were already sensitized to stress prevention. However, even in this informed and engaged group, the use in terms of fidelity and penetration was low, although System P was accepted by users and received good ratings for usability and appropriateness. Users were predominantly female, had a high educational status and good mental health. The high number of female participants is particularly striking in the context of MSE, where the proportion of female managers is usually low [68, 69]. However, the gender proportion is in line with the strongly represented health sector, which is generally more familiar with health and prevention measures [24].

Compared to other implementation studies, the adoption rate of System P among the MSE was quite low [18]. Yet, in relation to other communication strategies used during the unstructured approach (e.g., social media campaign), the conversion rate (from website visits to registrations) for e-mail contact via the recruitment partners was higher and compares well to other studies in small enterprises [70]. Accordingly, communication via intercorporate stakeholders seems to be generally suitable for the target group, but the appropriate communication strategy alone did not lead to the desired adoption of the intervention.

The MSE that decided to adopt System P were mainly from the health sector and professional, scientific and technical services and already sensitized to the issue of stress prevention (i.e., they had already dealt with measures to reduce stress, some had even carried out a PRA). They also described a good working atmosphere for their organization. These higher levels of expertise are in line with Benning et al. [22], who describe that conducting a PRA does require some diagnostic skills and knowledge of work demands and health risks in order to interpret the results. This could be an obstacle for small companies from other sectors that do not have the necessary expertise, thus discouraging them from adopting and using System P without external support. Although further verification is needed, these results may indicate that the potential health benefit (and cost effectiveness) of web-based interventions in the setting of MSE is low, since it is precisely companies that already have good working conditions and a commitment to stress prevention that implement them.

The managers and employees of MSE who adopted System P also showed a high affinity for technology and a high readiness for change. This confirms that the characteristics of the individuals involved, such as abilities and motivation [71] and attitude towards the intervention [72], contribute to the decision to implement an intervention. Other characteristics, such as general self-efficacy, might also play a role and should be further investigated [22, 23].

From the non-responder survey, we know that some MSE did not adopt System P because other business aspects were prioritized over the introduction of stress prevention or because there was no time (inner setting). This suggests that there is a lack of knowledge and conviction about the benefits of stress prevention measures and that existing legal regulations and scientific recommendations (outer setting) do not sufficiently motivate MSE to deal with the topic of stress prevention [22, 23]. The qualitative results underline the statements of the non-responders, as they indicate that stress prevention is not a priority in MSE. It is not clear whether the low priority of stress prevention results from a lack of intrinsic motivation of MSE (who did not adopt System P) or inhibiting external circumstances [26] such as coping with day-to-day business due to the shortage of skilled workers [27] or a combination of both.

When we explore the actual use of the individual components of System P by the registered MSE (research question three), the results reveal that even the sensitized and committed sample did not implement System P as intended. The targeted penetration rate was achieved in just three MSE. Moreover, not all employees of the participating MSE were invited to System P by their managers. A potential explanation for this may be that MSE find it complex to introduce an extensive and structural intervention for several people at the same time [24]. This is supported by the finding that the MSE in System P tend to initially introduce the intervention to only some of the employees (e.g., in individual departments), as indicated by the usage data of the PRA. Another reason why MSE managers did not invite all employees to System P could be difficulties in understanding the technical procedure of inviting employees. However, this is contradicted by the good evaluations of the usability of System P in the follow-up survey. It is also conceivable that the MSE consider the estimated benefit of System P or the relative advantage of the intervention to be too low [22, 31], or that the managers of the MSE consider prevention to be the personal responsibility of the employees [32].

During the study period, only two MSE carried out an analysis of possible work-related risk factors (step 1 of the PRA) and looked at the results. There was no introduction of appropriate countermeasures (only the effort to implement a measure in one MSE), which is in line with previous studies among MSE [73]. It is possible that managers of MSE prefer to deal with work-related stress in an informal way, outside the formalized framework of workplace health management [14, 74]. They may not see a real connection between their own practical work and PRA or perceive PRA as something imposed on them by others. This could be exacerbated by the stigma still attached to mental health in MSE and the consequent negative image of PRA [25]. Another explanation for the low usage of the PRA could be the perceived indirect costs. Stakeholders indicated that they expect a time-consuming communication effort accompanying the implementation of these stress prevention interventions. At the same time, the actual usage data from System P show that MSE spent only a very limited amount of time with the PRA. Consequently, there seems to be a gap between perceived and actual time investment, and between the direct and indirect costs of implementation. The reasons for this can be manifold, possibly the perception of time is distorted in stressful everyday life or the calculations include internal thoughts and discussions with the team.

The qualitative results indicate that managers need to be able to communicate the benefits of the PRA to their staff and that they may lack the specific knowledge of workplace stress prevention to be able to use System P appropriately without an external expert. This may be linked to concerns about the possible consequences of PRA, which may lead to unrest in the organization, unrealistic expectations or resistance that has to be dealt with [75, 76]. More offline time investments for communication, participation and change management within the MSE might be needed, which may itself become an additional barrier according to the qualitative results. Beck & Lehnhardt [17] also argued that increased contact with professional Occupational Safety and Health (OSH) experts by companies may help improve the use of stress prevention interventions. The use of the SMT also fell short of expectations based on prior studies which showed a higher compliance of participants with the program [37, 41]. Even though the training is available online and can be paused at any time, the usage data show that the participants did not spend much time with the training. It emerged from the qualitative results that managers have to provide their employees with the appropriate time to use System P. This time requirement in turn represents an additional investment that is less easy to compensate for in MSE than in larger companies [26]. In contrast to this argument, the participating MSE showed a high readiness for change and a good working atmosphere, which generally describes a good inner setting for the introduction of an intervention [22, 23]. However, personal characteristics such as learning style, seniority or values of the users may lead to the SMT not being used to its full extent [71].

System P was also designed to increase the adaptability of measures to the specific needs of MSE [25]. In response to our final research question (How do stakeholders perceive System P? ), the web-based intervention was described as appropriate for MSE, especially for those who already work with digital technologies. Overall, this fits with the stated affinity for technology of managers and employees in System P, who are generally open to engaging with new digital technologies. It can only be speculated that the openness to new digital technologies is less pronounced in other sectors that are hardly or not at all represented in the present sample, such as craft enterprises, and thus hindered the adoption of System P [77]. There is some evidence in the literature, which shows that the level of digitalization in MSE is less advanced than in larger companies [78, 79]. In addition, digital technologies are primarily used in the context of everyday working tasks (e.g., e-mail communication) and hardly or not at all when it comes to improve work processes [78].

### Implications

Overall, the results of the implementation of System P show that in order to adopt and fully integrate stress prevention in the daily routine of MSE, awareness and knowledge of stress prevention must continue to increase so that the issue becomes more of a priority in organizations than it has been so far. In any case, it was clear from our study that a state-of-the-art web-based system alone had little effect on the ability of MSE to initiate and carry out stress prevention interventions.

Certainly, some good and perhaps unconscious efforts to reduce stress prevention are already taking place in MSE on an informal level [74], but in our opinion this does not replace systematic stress prevention. However, for a concrete and comprehensive establishment of stress prevention in MSE, it will be necessary to demonstrate the actual benefit or return on investment. This is because the indirect costs (e.g., time investment) to adopt and use an intervention such as System P (despite the high flexibility offered by a web-based solution) appear to be too high for MSE, as they seem to have already reached the limit of their capacity with their day-to-day business due to external circumstances such as a shortage of skilled workers or inflation [24, 26, 27]. The MSE which have been active in System P are likely to perceive a lower cost and lower risk associated with the introduction of a new intervention due to their good working climate, the employees’ willingness for change and the already increased knowledge of stress prevention and are therefore higher willingness to engage in System P than other MSE. For other MSE, one could consider stronger regulations or more control mechanisms as facilitators to implement stress prevention. Yet, these measures are likely to increase the pressure on small companies and might potentially lead to additional resistance [80]. Instead, increased support for MSE at various levels (e.g., instrumental, financial, bureaucratic relief) could be a more effective strategy. The use of web-based interventions could be accompanied and guided by professional OSH experts, given that the lack of knowledge about stress prevention and the mechanisms for implementing an intervention such as System P (including sensitive communication and careful interpretation of the results) also appears to be a criterion for non-adoption, especially outside the health and technical services sectors. Future research on stress prevention in MSE should therefore focus more on the return on investment for MSE and allow for a much greater degree of freedom in implementation and documentation as well as better (external) support for the integration with general entrepreneurial and management tasks.

### Strengths and limitations

This study is one of the first systematic evaluations of the implementation of stress prevention in the setting of MSE and it has several strengths. First of all, we used many different communication strategies and media channels to reach the target group and were therefore able to compare their effectiveness. This provided insight for practitioners and public health stakeholders who want to target MSE (e.g., that a more targeted and personal communication is overall more effective than mass or social media campaigns).

Secondly, we worked together with intercorporate stakeholders who are in direct exchange with MSE from various sectors and who were mostly mentioned as the main source of information on prevention issues by the users of System P. Researchers should continue to work together with these stakeholders when developing/adapting interventions and choosing communication approaches. However, personal contact, which was most effective, is also the most time-consuming type of recruitment and increases the indirect costs on both provider and user side.

Finally, we combined the quantitative results with qualitative data to gain specific and further insights into the perceptions and assessments of System P among the target group and stakeholders. This approach fills knowledge gaps about the implementation process in MSE and answers some questions about barriers to the implementation process raised by the low dose and fidelity in the first part of the study.

The study also has several limitations. It was originally set up with three measurement points but due to the long recruitment phase and slow uptake, T3 (another 6 months later) could not be completed. Therefore, this study lacks information on the sustainability of the implementation process. Overall, the recruitment took longer than expected and the drop-out between measurements was high. Therefore, several of the originally planned comparisons (pre-post measurements) were not possible within the given timeframe and no conclusions on the real-world effectiveness of the interventions could be made. One possible explanation for the drop-out among managers and employees is that the recruitment took place during the ongoing COVID-19 pandemic, which challenged companies in a fundamental way [81].

Due to the small sample size, the results may not be representative for all MSE. However, because the intervention was rolled out across the country and through many media channels, we can assume that theoretically a large proportion of the target group was aware of the intervention.

Moreover, due to the extensive amount of measured outcomes and control variables the questionnaires within the implementation study were quite long. This could have stopped some interested users from participating further after the registration. To take this into account, we often used short versions or single item measures instead of full questionnaires. Therefore, the validity and reliability is restricted. Future studies should use fully validated instruments to measure a selection of process outcomes when possible (e.g. ORIC for Organizational Readiness for Implementing Change [82] and adapt them for the MSE setting.

Finally, the interview data only captured perceived suitability and usefulness as well as potential barriers from the perspective of MSE managers and intercorporate stakeholders. In light of the convenience sampling, the qualitative results might not be fully representative of all MSE in Germany. Ideally, we would have interviewed actual users of System P at the start and again during or after the implementation period, but this was not possible without a study extension, given the slow and difficult recruitment. Due to the high drop-out, conclusions on the actual suitability and usefulness are based on a few quantitative measures from T2 and cannot be generalized. Future studies should include the view of employees on usefulness and fit and also assess how the perception might change over time during use.

## Conclusion

In conclusion, the results of the present study point towards several barriers to the implementation process of stress prevention interventions among MSE. Despite of a high general acceptance of web-based interventions, the overall complexity and perceived (indirect) costs, e.g. time investment, make it less feasible for small enterprises without external help. This results in minimal use of the intervention. Considering the potentially high long-term costs of stress-related illnesses for MSE, communication efforts should be increased and additional support from intercorporate stakeholders who are already sensitized and well-informed is necessary to facilitate the implementation process.

### Electronic supplementary material

Below is the link to the electronic supplementary material.

Supplementary Material 1

Supplementary Material 2

Supplementary Material 3

Supplementary Material 4

Supplementary Material 5

Supplementary Material 6

Supplementary Material 7

## Data Availability

No datasets were generated or analysed during the current study.

## Footnotes

1 Additional visits to the project websites from other sources explain the discrepancies between visits due to the standardized e-mails from R1 reported in Fig. 1 (*n* = 157) and total visits in December 2021 and January 2022 reported in Fig. 2 (*n* = 467).

2 The reported visits to the project website only refer to the number of **tracked visits** per month for those who enabled the necessary cookies in their browser. The real number of visits is assumed to be higher.

## Abbreviations

GDA: Gemeinsame Deutsche Arbeitsschutzstrategie (Joint German Occupational Safety and Health Strategy)
DESTATIS: Statistisches Bundesamt (German Federal Statistical Office)
MSE: Micro and Small-Sized Enterprises
PRA: Psychosocial Risk Assessment
SMT: Stress Management Training
OSH: Occupational Safety and Health
