# Supplementary material for: Web-based occupational stress prevention in German micro- and small-sized enterprises – process evaluation results of an implementation study
Source: BMC Public Health. 2024 Jun 17;24:1618. doi: 10.1186/s12889-024-19102-8 (PMC11184923; doi:10.1186/s12889-024-19102-8)
Supplement: Supplementary file 1 — Supplementary Material 1 [file 12889_2024_19102_MOESM1_ESM.docx]

# Module 1

## Workplace-Check (Psychosocial Risk Assessment, PRA)

System P`s Workplace-Check (WPC) includes a psychosocial risk assessment, which can be carried out independently and without professional expertise. The WPC consists of three steps: (1) Preparation, (2) Stress-Analysis and countermeasures, and (3) Evaluation. Due to different working conditions, one WPC per area of activity should be conducted to obtain the most meaningful results.

### (1) Preparation

On the Preparation page, employers create questionnaires to measure the level of stress and analyze current working conditions in the company. They designate different areas of work within the company and create separate online-questionnaires for each defined area, as each area may have specific working conditions. If only few employees belong to an area of work, it is recommended to combine similar areas of work or to create a survey for the entire company instead.

While creating a survey on System P, employers can choose question blocks from the integrated question pool and/or create individual questions. The system’s questionnaire pool contains an extensive range of pre-formulated questions covering all areas defined by the German Health and Safety Association (“Gemeinsame Deutsche Arbeitsschutzstrategie” (GDA)) as well as additional questions concerning new forms of work, including, for example the impact of the COVID pandemic. Every question block consists of a filtering question (e.g., Is there any stress?), one question concerning the origins of the stress (e.g., What causes the stress?), and one question regarding possible countermeasures (e.g., How can the stress be reduced?). By default, the system preselects nine question blocks covering the most significant stress factors, according to researchers and the GDA.

To invite employees to participate in the survey, employers can assign them to the different work areas. In this way, employees will receive an automatically generated e-mail with an individual link to the questionnaire. Alternatively, the employer can simply share the link to the survey. If a company has only a few employees (e.g., less than five employees), the WPC can be done by the employer; in this case, the employer fills out the questionnaire himself or herself, ideally in cooperation with the employees.

### (2) Stress-analysis and countermeasures

Once a survey has been finished, the results are shown in the Stress-Analysis and Countermeasures page. Here, the results are presented both graphically and in percentage frequencies. The colours in the bar chart illustrate in green, yellow, and red the stress level results for each question block. The results of the questions concerning causes for stress and possible countermeasures are shown below the bar charts. In addition, System P provides countermeasure suggestions by experts for each question block. Within each question block, employers can plan work and safety measures and document the implementation progress of these countermeasures. The page offers different sorting options, for example, to display the question blocks with the highest stress levels at the top.

A short questionnaire can be activated by mouse-klick, in which employers can draw feedback concerning possible countermeasures from their employees. This step aims at employee involvement: Employees are given the opportunity for active participation by indicating their preferences for and acceptance of possible countermeasures before the measures are implemented.

### (3) Evaluation

On the Evaluation page, the implemented countermeasures can be evaluated. Employers can start an evaluation questionnaire and later review the results. This questionnaire is addressed to the employees who participated in the first stress analysis questionnaire (Step 1: Preparation). The page lists the implemented countermeasures and aims at analyzing their effectiveness: Did the implemented measure improve the specific situation? In a free text field, employees can describe their suggestions to improve the measure further.

Additionally, employers can document on this page why proposed measures have not (yet) been implemented. An automatic reminder helps employees to not forget about the evaluation. All results of the stress-analysis, derived countermeasures, and the evaluation are documented in System P and can be exported as PDFs.

# Module 2

## Web-based stress management training “GET.ON Stress”

The web-based online stress prevention training “GET.ON Stress” is available to employers as well as employees after having registered at System P. The training consists of seven sessions that participants should work on following a weekly schedule (table 1). Each session consists of general information, interactive exercises, prototype training participants (so called *personas*– who represent different stressed employee groups), quizzes, audio and video files, and downloadable work sheets. The training aims to enhance two major strategies of stress coping: problem-solving and emotion regulation. In addition, at the end of sessions 2 to 6, users can choose to obtain extra information and perform short exercises about the following common stress-related topics: time management, rumination and worrying, psychological detachment from work, sleep hygiene, sleeping habit rhythm and regularity, nutrition and exercise, organization of breaks during work, and social support.

| **Table 1. Contents of the web-based stress management training (SMT)** | |
| --- | --- |
| **Session^a^** | **Intervention content** |
| 1 | Psychoeducation on stress and coping competencies  Enhancement of pleasant activities |
| 2 | Problem-solving I – identifying and differentiating solvable and unsolvable problems; developing an initial problem-solving plan  Information and exercises on selected topics, which users can self-select^b^ |
| 3 | Problem-solving II – self-evaluating the problem-solving plan; adapting or developing a new problem-solving plan  Information and exercises on selected topics, which users can self-select^b^ |
| 4 | Emotion regulation I – progressive muscle relaxation  Information and exercises on self-selected topics^b^ |
| 5 | Emotion regulation II – acceptance and tolerance of (negative) emotions  Information and exercises on self-selected topics^b^ |
| 6 | Emotion regulation III – effective self-support in times of stress  Information and exercises on self-selected topics^b^ |
| 7 | Developing a stress-coping plan for the future |
| Note: ^a^ each session lasts approximately 45 to 90 minutes; ^b^ optional exercises will cover the topics of time management, rumination and worrying, psychological detachment from work, sleep hygiene, the rhythm and regularity of sleeping habits, nutrition and exercise, organization of breaks during work, and social support | |

The following descriptions of the training content have been derived from the study protocol from Heber et al. (2013).

Session 1: In the first session, the participants will be provided with psycho-educational information about stress based on Lazarus’ transactional model of stress, which includes emotion-focused and problem-focused coping strategies. Following a video introduction to the basic information about stress and coping methods, an interactive quiz will be presented to equip participants with general knowledge of the most appropriate coping strategies for common problem situations. The participants will identify their personal stressors and define their goals and motivations during the training. Furthermore, participants will be asked to choose one positive activity each day.

Session 2–3: In sessions 2 and 3, the participants will work on their problem-solving skills. This module is based on problem solving therapy. The participants will learn a systematic six-step problem-solving method that can be applied to their individual problems. This method has already been adopted in other web-based studies. Typical scenarios involving work-related stress will be presented. The participants will fill out their own six-step procedures and use their personal solutions in the time between the sessions. In session 3, the participants will have the opportunity to either work on the same problem as in the previous session or choose a new problem.

Session 4–6: In sessions 4 to 6, the participants will work on the emotion regulation module. The emotion regulation techniques are based on the Affect Regulation Training (ART) and include muscle and breathing relaxation, acceptance and tolerance of emotions, and effective self-support. ART has been shown to be effective as an additional treatment component in cognitive-behavioural treatment for depression and also as a stand-alone intervention in at risk-populations (i.e., police officers). In each session, one strategy will be taught, and the participants will practice that strategy with a 15-minute audio file according to the instruction of an expert. The techniques will be introduced using examples of typical emotional reactions related to work contexts. Video files explaining the psychological background will be displayed. The participants will be asked to listen to the appropriate 15-minute audio file on a daily basis and will be provided with downloadable mp3 files.

Session 7: In the last session, the participants will be asked to reassess their goals for the training. Furthermore, they will identify their personal warning signs for stress. Additionally, they will be asked to strengthen something important in their lives and write a letter to themselves about how they imagine their life will be after four weeks of applying the stress-management methods they have been trained in.

In addition to training content, participants can receive written feedback from an e-coach on their exercises after each training session.

# Module 3

## Stress-encyclopedia

System P provides users with a stress-encyclopaedia encompassing background information on the topic of stress. The **Stress-Encyclopedia** is divided into the themes of health protection and prevention, stress in general, as well as stress at the workplace. It explains technical terms from research and the field of health protection and introduces scientific models regarding the origins of stress.

# Module 4

## Exchange all around the topic of stress (FAQ & Forum)

Any questions regarding the system and the implementation of stress prevention at the workplace can be asked by users in module 4. Frequently Asked Questions will be answered in the FAQ section. Employers also have access to a forum where they can exchange their experiences with other employers.

- The **FAQ** (frequently asked questions) provides answers to questions that might arise with users of the stress prevention platform System P. The FAQ topics cover System P itself, data privacy protection, the implementation of the program in the workplace, the use of System P, the psychosocial risk assessment, as well as motivating users and corporate strategy.
- In the **moderated forum,** employers can exchange their experiences with System P, ask questions, or answer those of others. All posts will only be published after verification by a moderator to ensure anonymity.

# Literature

Heber, E., Ebert, D. D., Lehr, D., Nobis, S., Berking, M., & Riper, H. (2013). Efficacy and cost-effectiveness of a web-based and mobile stress-management intervention for employees: design of a randomized controlled trial. BMC Public Health, 13(1), 655. <https://doi.org/10.1186/1471-2458-13-655>
