# Supplementary material for: Web-based occupational stress prevention in German micro- and small-sized enterprises – process evaluation results of an implementation study
Source: BMC Public Health. 2024 Jun 17;24:1618. doi: 10.1186/s12889-024-19102-8 (PMC11184923; doi:10.1186/s12889-024-19102-8)

# Standardized E-Mail

### **Subject:** Digital Stress Prevention at Work – Free Access for Small Enterprises

Dear Ladies and Gentlemen/Dear Ms. X,

we are pleased to present you an offer of the Heinrich-Heine-University Düsseldorf: "**System P**" is an online platform for micro- and small-enterprises - and accessible to you free of charge.

The online platform System P supports you and your employees to manage stress effectively. By using System P, you comply with the requirements of the Occupational Health and Safety Act, create the basis for a healthy work environment. and ensure the productivity of your company.

To learn more about System P and the benefits of stress prevention for your company, we cordially invite you to the **information event: "Stress prevention in small enterprises - Simple, digital, and free of charge with System P"**.

The event will take place on Thursday, December 16, 2021 at 5pm or January 21, 2022 at 3pm.

**More information about the offer, the online platform, and the event can be found at** [**www.stressprävention-im-betrieb.de**](http://www.stressprävention-im-betrieb.de)

**If** have any questions, please feel free to contact the research team at Heinrich Heine University Düsseldorf directly ([system-p@hhu.de](mailto:system-p@hhu.de)).

We look forward to your participation!

Kind regards

NAME / INSTITUTION

The online platform was developed by three German universities within the research project "PragmatiKK". The project is funded by the German Federal Ministry of Education and Research as part of the " The Future of Work” program.


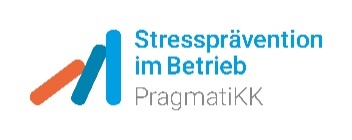

Supplement: Supplementary file 2 — Supplementary Material 2 [file 12889_2024_19102_MOESM2_ESM.docx]
