# Supplementary material for: Web-based occupational stress prevention in German micro- and small-sized enterprises – process evaluation results of an implementation study
Source: BMC Public Health. 2024 Jun 17;24:1618. doi: 10.1186/s12889-024-19102-8 (PMC11184923; doi:10.1186/s12889-024-19102-8)

## Stress Prevention Forum

Stress prevention programs are also primarily about what individual enterprises make of them. That's why it's important to share experiences and learn from each other. The forum gives you the opportunity to do this in moderated form. This means that each contribution is first checked by the moderators to ensure that it complies with the forum's rules of conduct. This includes, for example, not using clear names or a respectful tone. If this is the case, the moderation activates the post, which can then be viewed by all forum participants.


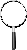
What would you like to know?

For more information about the Forum, see <https://www.stresspraevention-im-betrieb.de/>


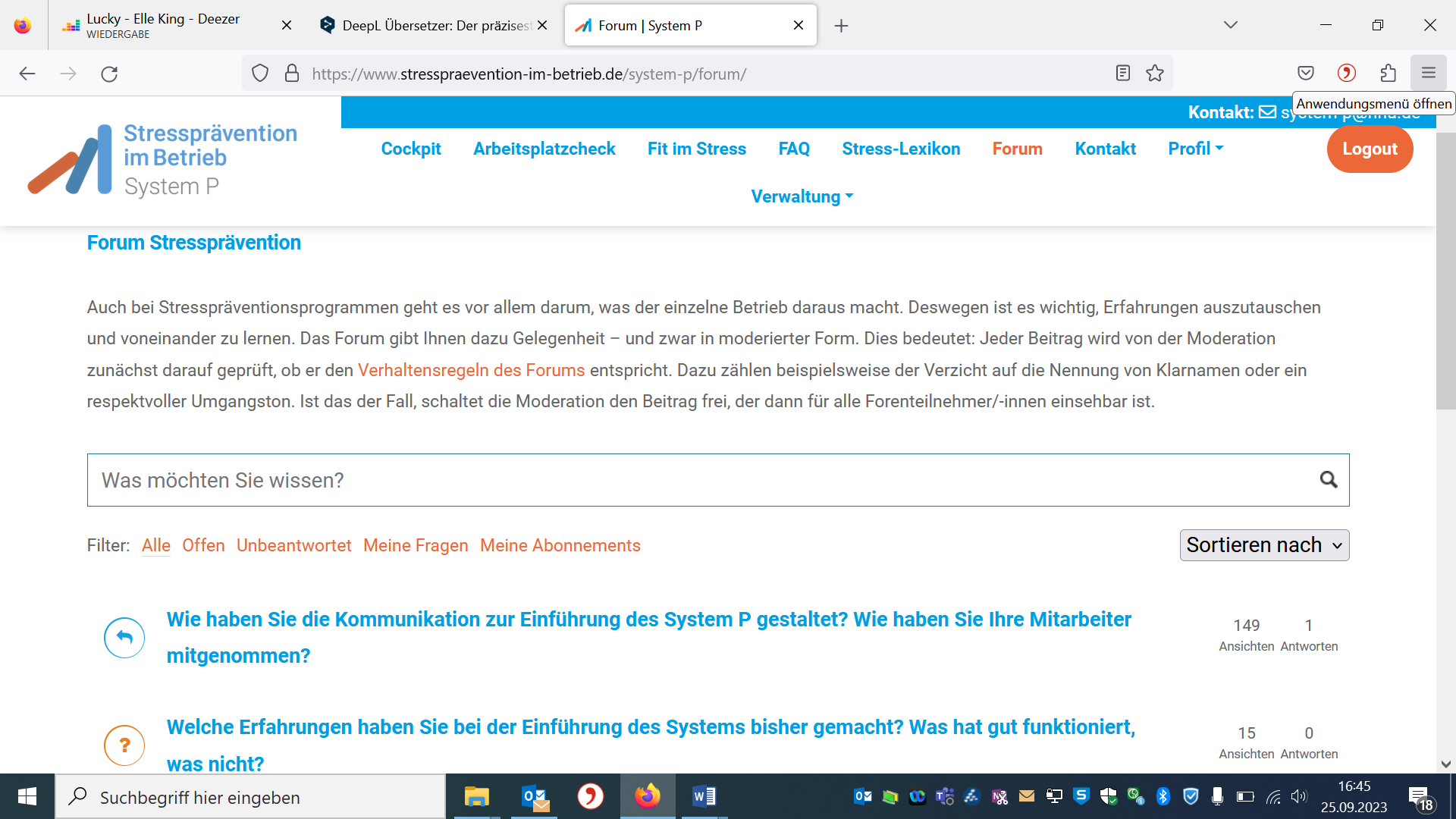

Supplement: Supplementary file 3 — Supplementary Material 3 [file 12889_2024_19102_MOESM3_ESM.docx]
