# Supplementary material for: Web-based occupational stress prevention in German micro- and small-sized enterprises – process evaluation results of an implementation study
Source: BMC Public Health. 2024 Jun 17;24:1618. doi: 10.1186/s12889-024-19102-8 (PMC11184923; doi:10.1186/s12889-024-19102-8)

# System P FAQ

## **FAQ – Questions and answers**

What is System P? How long does the training take? What technical requirements do I need? What should be considered during the introduction? We answer the most important questions about the stress prevention program - all other questions you can ask us via our contact form.

## About System P - Stress prevention in the enterprise

### What is System P?


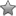

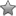

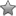

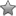
(No ratings yet)
***How useful do you find this information?***


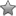


The System P stress prevention platform was developed specifically for use in micro- and small-businesses. There, time and resources are usually scarce. In addition, there is often a lack of knowledge about the connections between stressful situations, stress, and illness. System P combines the risk assessment of mental stress with an individual stress prevention program and provides detailed information on the topic of stress. All modules are available online - and thus independent of time and location.

Who is System P suitable for?


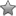

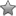

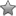

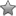

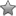
(No ratings yet)
***How useful do you find this information?***

System P is aimed at micro and small enterprises with up to 49 employees. During the trial phase, only companies in this group should participate. The system is suitable for all companies that have not yet used a stress prevention program.

What are the benefits of participating in System P?


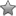

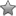

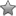

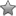

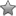
(No ratings yet)
***How useful do you find this information?***

As an employer, you will find out what stress risks exist in your enterprise. With the workplace check, you also comply with the legal requirements for risk assessment of mental stress and meet the requirements for implementing a professional stress prevention program. You will receive a certificate for this. Consistent stress prevention supports you in making your company an attractive employer. With the stress prevention training "Fit in Stress" you train individually how to recognize and deal with stress in your everyday work. This reduces work-related stress, increases your well-being, and helps you to get health risks under control.

How much time should we allow?


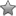

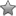

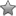

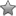

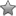
(No ratings yet)
***How useful do you find this information?***

The stress prevention program is introduced gradually in the company, usually over a period of several months. For the two main modules, the workplace check and the stress prevention training, it can be roughly stated:

Workplace check: basically, the effort varies greatly depending on the needs and preferences of your company. Answering the stress analysis in the form of the standard questionnaire takes about 5 minutes, the fully comprehensive version takes about 45 minutes.

Stress management training: The "Fit in Stress" training includes 7 units that you can complete entirely at your own pace. To get the most out of the training, we recommend that you complete one training session per week and take about 45 minutes for each session. Between training sessions, you will have the opportunity to try out what you have learned in your everyday life.

The baseline and final surveys each take about 15 minutes.

Why is System P available only online?


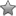

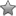

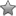

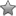

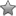
(No ratings yet)
***How useful do you find this information?***

System P is designed to enable small companies to prevent stress and to do so particularly quickly, flexibly, and anonymously for employees. The online format combines these advantages:

- Problems that employees do not want to or cannot communicate directly to their superiors are forwarded anonymously. Supervisors learn of difficulties without being able to trace them back to the individual employee.
- They have the opportunity to attend training sessions tailored to their specific needs. The system enables all members of the company, supervisors and employees, to prevent stress.
- The system shows an overview of where there is a need for action in stress prevention. This can help to develop individual solutions.

Is the use of System P free?


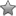

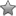

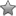

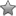

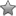
(No ratings yet)
***How useful do you find this information?***

System P is currently free of charge for organizations.

Which technical requirements do I need?


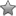

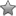

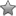

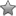

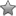
(No ratings yet)
***How useful do you find this information?***

The System P stress prevention platform is available exclusively online. To participate, you need Internet access, an end device such as a computer, tablet, or smartphone, and - ideally - a quiet place to work in a concentrated manner. The system works best via the Mozilla Firefox and Google Chrome browsers. We also recommend performing the workplace check on the computer so that all functions and questions can be reviewed.

Who developed System P?


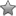

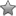

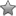

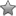

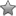
(No ratings yet)
***How useful do you find this information?***

The stress prevention platform, System P, is the subject and result of the PragmatiKK research project. Scientists from five university institutes, as well as practice partners, are involved. The research project examines what suitable stress prevention should look like for micro- and small-enterprises and develops an online offering for this target group.

What practical and scientific qualifications do the contact persons have?


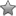

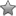

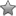

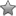

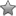
(No ratings yet)
***How useful do you find this information?***

The PragmatiKK team consists of experts with practical and scientific experience in the fields of occupational psychology and medicine:

- Industrial and organizational psychology
- Health promotion and prevention
- Psychological workload

For more information about the FAQ, see <https://www.stresspraevention-im-betrieb.de/>


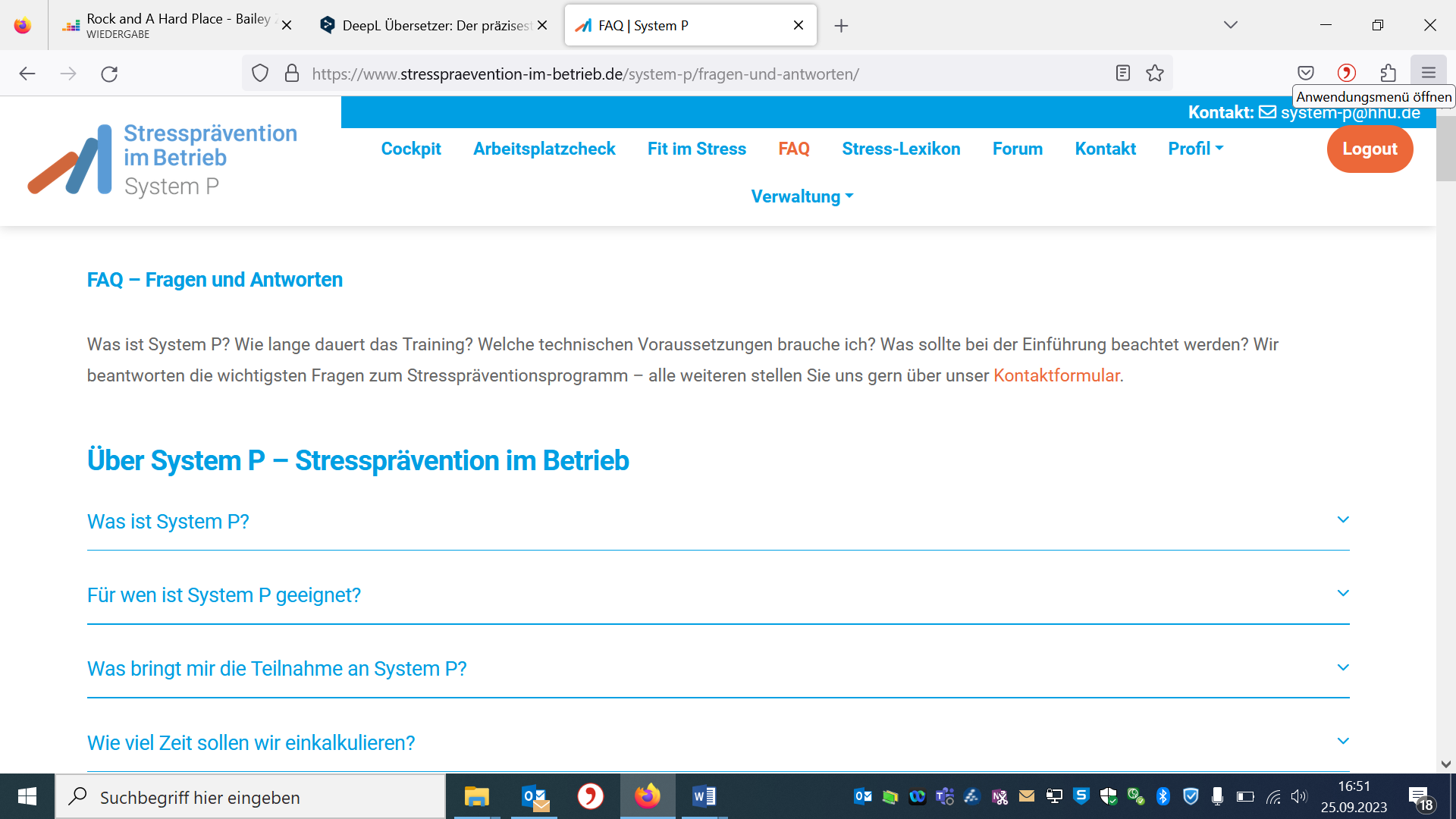

Supplement: Supplementary file 4 — Supplementary Material 4 [file 12889_2024_19102_MOESM4_ESM.docx]
