# Supplementary figures and images for: Web-based occupational stress prevention in German micro- and small-sized enterprises – process evaluation results of an implementation study
Source: BMC Public Health. 2024 Jun 17;24:1618. doi: 10.1186/s12889-024-19102-8 (PMC11184923; doi:10.1186/s12889-024-19102-8)

# Social-media post

### Example images


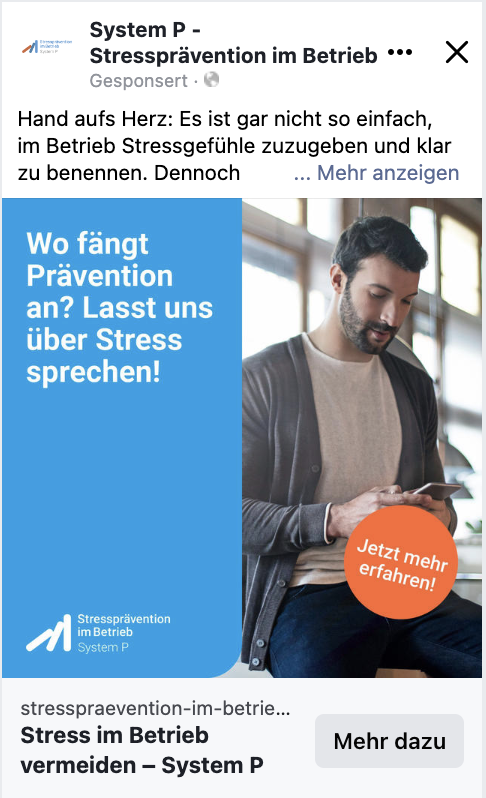


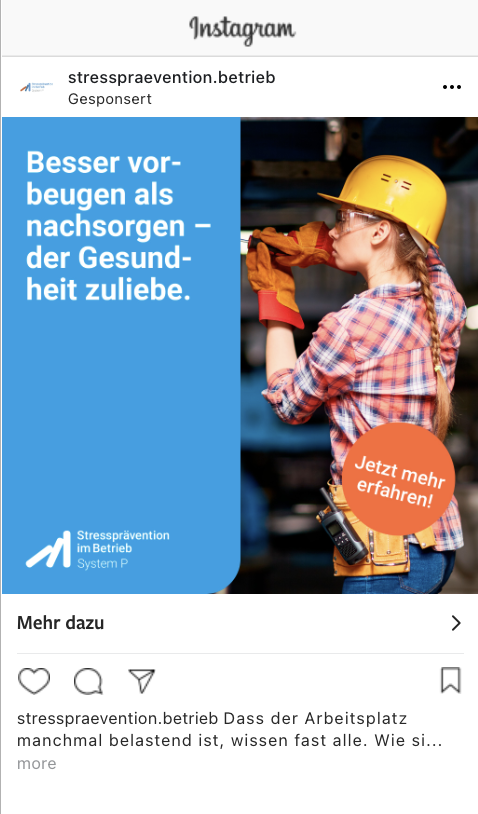


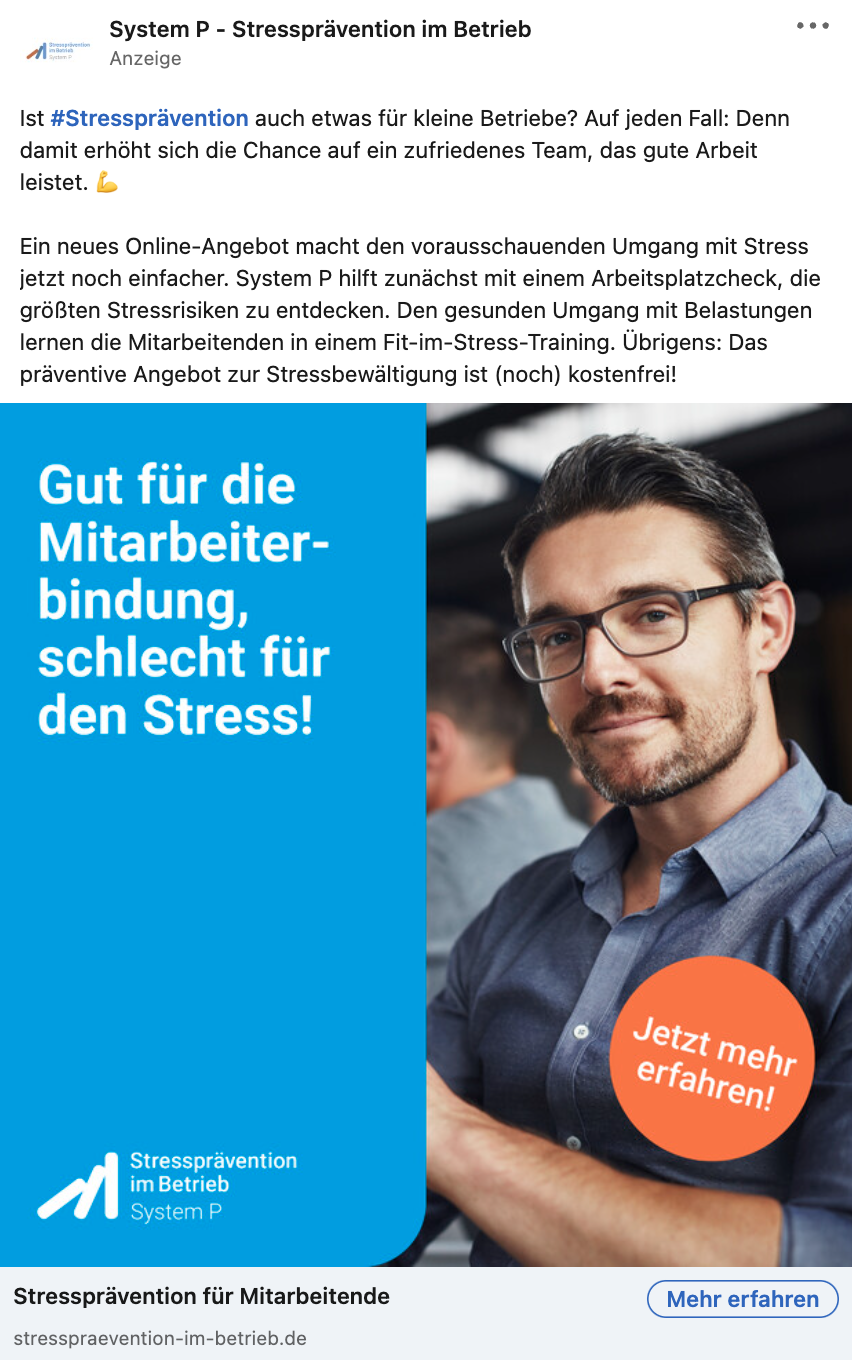

Supplement: Supplementary file 5 — Supplementary Material 5 [file 12889_2024_19102_MOESM5_ESM.docx]
