# Supplementary material for: Web-based occupational stress prevention in German micro- and small-sized enterprises – process evaluation results of an implementation study
Source: BMC Public Health. 2024 Jun 17;24:1618. doi: 10.1186/s12889-024-19102-8 (PMC11184923; doi:10.1186/s12889-024-19102-8)
Supplement: Supplementary file 6 — Supplementary Material 6 [file 12889_2024_19102_MOESM6_ESM.pdf]

Preparation

Guide
Recorder (check rechargeable battery/battery)
Paper/pen for notes
Study information/consent form (if not sent in advance, have it signed and sent by e-mail)
Preliminary questionnaire (if not completed in advance)
Share link to videos in chat<br>- https://stresspraevention-im-betrieb.de/system-p/interview<br>- be prepared to share the videos in an emergency (already have them open in Media Player)
Login to System P to briefly display Cockpit and FAQ, Stress Lexicon and Forum

Overview

Block 1: Introduction	10-15 minutes
Block 2: Impression system P	5-10 minutes (incl. video 1:03 min.)
Block 3: Workplace check	10-15 minutes (incl. video 6.24 min.)
Block 4: Online training	10 minutes (incl. video 3:28 min.)
Block 5: FAQ, stress lexicon and forum	5 minutes
Block 6: Feasibility and utilisation System P	5-10 minutes
Block 7: Conclusion	5 minutes
Total	**50 - 70 minutes**

Block 1: Introduction

Presentation

- Thank you for your willingness to talk
- Introduction of the interviewer
- Today's interview will focus on your experience with the System P stress prevention platform

Procedure

- The interview will last about an hour
- I have brought a few questions with me that I will ask you one by one. As I don't know all the questions off by heart, I will **sometimes read out questions or look at my documents and make notes on them** - don't let this confuse you.
- An important note in advance: There **are no right or wrong answers**, we are primarily interested in your point of view.
- During the course of the interview, I will ask you to watch **3 short videos on System P** via the link provided in the chat. Please don't be confused by the abrupt transitions - the individual videos have been edited together from a longer video. So that you can watch the respective video in peace, I will switch off my video in the meantime.
- I'll keep an eye on the time so that we stay roughly within the time frame.

Data protection and recording

- In order to be able to analyse the content of the interview retrospectively, we will **record** the **conversation** - only the sound will be recorded.
    - *Show device*!
    - *Alternatively, use the video conferencing tool*: You will soon hear a message that the recording is starting.
- When the data is written down, it is anonymised, i.e. all personal details, e.g. names, are made unrecognisable.
- We had already sent you a few documents in advance (the study information, the **declaration of consent and the preliminary questionnaire**). Have you read and signed or completed the documents or do you have any questions?
- Do you have any further questions before we start?
    - *Start recording*

Introduction of company and interviewee

I would like to start by asking you to briefly introduce yourself and your organisation.

What does your organisation do?

- Are there special support programmes for small companies?
- Are there any offers in the area of stress prevention?

What is your role in your organisation?

Block 2: Impression system P

Introduction

- The aim of our discussion today is to talk about your impressions of System P.
- As mentioned at the beginning of our conversation, I would now like to ask you to watch the first video sequence (trailer) via the link shared in the chat. I would then like to talk to you about your first impression of System P.

Scenario 1: Video not viewed	Scenario 2: Video viewed
→ *Show 1st video sequence (trailer) (1:03 Min.) [switch off own video*]<br>→ *[Switch on your own video again]*<br>*-> Questions "1st impression"*	• When did you watch the video?<br>• Do you still remember this well?<br>→ *if the answer is yes: -> Questions "1st impression"*<br>→ *if negative: -> Scenario 1*

- Please tell us in detail about both your positive and negative impressions. Your answers will help us to further customise the system to the needs of small companies.

First impression

Let's start in general terms: What is your first impression of System P?

- What did you particularly like?
- What did you notice that was negative?

How would you describe the P system to a colleague?

- What is the goal of System P in your eyes?
- What do you think are the most important functions?

Module overview

System P consists of a total of four modules, which you can see in the cockpit on the start page.

- **Workplace check**
- **Online training "Fit under stress"**
- **FAQ** with information on the introduction and use of the system as well as a knowledge database in the form of the **stress lexicon**
- Opportunities to share practical experience with the **forum**

Block 3: Workplace check

Introduction

- Now I would like to discuss the two main modules individually and start with the workplace check.
- Firstly, I would now like to ask you to watch the second video sequence (workplace check) via the link shared in the chat.

Scenario 1: Video not viewed	Scenario 2: Video viewed
→ *2nd video sequence (workplace check) show (6:24 min.)*<br>*[Switch off your own video]*<br>→ *[Switch on your own video again!]*<br>*-> Questions about the workplace check*	• When did you watch the video?<br>• Do you still remember this well?<br>→ *if the answer is yes: -> Questions about the workplace check*<br>→ *if negative: -> Scenario 1*

How would you describe the workplace check in your own words?

In your opinion, how well can the workplace check be implemented in small companies?

- What is easy to implement?
- What is difficult to realise?
- How do you rate the possibility of adapting the workplace check to the local needs of small companies?
- What would you like to change or what are you missing?
- How much time do you expect it will take for the companies?

In your opinion, how helpful/useful is the workplace check?

- To what extent do you believe that you can identify and change stresses in small companies through the check?
- How practicable is the workplace check for small companies?
- To what extent could the workplace check reduce stress levels in small companies?
- What other benefits do you expect from the workplace check?
- To what extent could the workplace check increase the workload in small companies?

The workplace check is a risk assessment of mental stress in accordance with the Occupational Health and Safety Act.

Have you already had experience with GBP?

[If you have experience with GBP]

How do you find the workplace check in comparison with other GBP offers?

What do you think of the term "workplace check"? Are there other terms that you find more appropriate?

What else did you notice during the workplace check?

Block 4: Fit under stress

Introduction

- Now I would like to talk about the online training programme Fit in Stress.
- I would now like to ask you to watch the third video sequence (training) via the link shared in the chat.

Scenario 1: Video not viewed	Scenario 2: Video viewed
→ *3rd video sequence (training) show (3:28 min.)*<br>*[Switch off your own video]*<br>→ *[Switch on your own video again!]*<br>*-> Questions about training*	• When did you watch the video?<br>• Do you still remember this well?<br>→ *if the answer is yes: -> Questions about the training*<br>→ *if negative: -> Scenario 1*

How would you describe the training in your own words?

How well do you think the training can be implemented in small companies?	
	- What is easy to implement?<br>- What is difficult to realise?<br>- What would you like to change or what are you missing?<br>- How reasonable do you find the time required?
**How helpful/useful is the training in your opinion?**	
	- To what extent do you believe that the training can improve the way we deal with stress?<br>- How practicable is the training for day-to-day work in small companies?<br>- To what extent could the training reduce stress levels in small companies?<br>- What other benefits do you expect from the training?<br>- To what extent could the training increase stress in small companies?
**Have you already had experience with other stress management training courses?**<br>*[If you have experience with other stress management training courses]*<br>**How do you find the training programme compared to other stress management training courses?**	
What else did you notice during training?	

Block 5: FAQ, stress lexicon and forum

Introduction

Now I would like to talk about the other services in System P: the FAQ, the stress encyclopaedia and the forum.

I will now share my screen and show you these offers in System P.

- The FAQ answers important questions about the system itself and the introduction of this tool.
- The stress lexicon provides further information on the topics of "stress" and "health protection".
- The forum enables an exchange with other company owners about the system.

How do you rate these additional services in the P system?

Stressprävention im Betrieb PragmatiKK

Block 6: Feasibility and utilisation System P (stakeholders)

Finally, I would like to talk to you again about your general impression of System P.

How well do you think the system can be implemented in small companies?

- What is easy to implement?
- **What is difficult to realise?**
- **What are you missing?**
- How do you rate the system's adaptability to local needs in small companies?
- **How do you rate the composition of the modules in the system?**
  - Would you want to change anything?
  - What aspects of behavioural prevention are missing?
- **How do you rate the design of the system?**
- How would you rate the time involved?

What benefits do you expect from System P?

- for small companies
- for their employees

To what extent can you imagine small companies using the platform?

Would you recommend the platform to micro and small entrepreneurs?

- Who would you recommend it to?
- Who doesn't?
- For what reasons?

System P is currently available to small companies free of charge until at least the end of this year. How do you rate the willingness of small companies to pay for a platform like this?

- How much do you think is appropriate?

Block 6: Feasibility and utilisation System P (managers)

How well do you think the system can be implemented in your company?

- What is easy to implement?
- **What is difficult to realise?**
- **What are you missing?**
- How would you rate the system's adaptability to your company's local requirements?
- **How do you rate the combination of modules in the system?**

  Would you want to change something? What is missing?
- **How do you rate the design of the system?**
- How would you rate the time involved?

What benefits do you expect from System P?

- for your company
- for your employees
- for you personally

System P is currently available to small companies free of charge until at least the end of this year. Would you be prepared to pay for a platform like this once our project is complete?

- How much approximately?

We have now talked a lot about your company. If you think about other small businesses - would you recommend the platform to other micro and small entrepreneurs?

- Who would you recommend it to?
- Who doesn't?
- For what reasons?

Stressprävention im Betrieb PragmatiKK

Block 7: Conclusion

Conclusion

- Final question: **What else have you noticed that we haven't talked about today?**
- If necessary, ask questions from the preliminary questionnaire
- Thanks for your time and interest
- Would you agree that I should contact you again if I have any further questions after today?
- Would you consider using quotes from this interview as testimonials to promote the tool?
    - If this is the case, we will come back to you with the details and you can withdraw your consent at any time
- Stop recording

Next steps

- In the next step, we will now transcribe the audio recording and analyse it anonymously together with other transcripts.
- Your answers will enable us to customise the platform even better to the needs of small businesses.

Last questions

- Do you have any questions about how to proceed or the project?
