# Supplementary material for: Web-based occupational stress prevention in German micro- and small-sized enterprises – process evaluation results of an implementation study
Source: BMC Public Health. 2024 Jun 17;24:1618. doi: 10.1186/s12889-024-19102-8 (PMC11184923; doi:10.1186/s12889-024-19102-8)
Supplement: Supplementary file 7 — Supplementary Material 7 [file 12889_2024_19102_MOESM7_ESM.pdf]

Consolidated criteria for reporting qualitative research (COREQ)

No	Item	Description
**Domain 1: Research team and reflexivity**		
Personal characteristics		
1.	Interviewer	Conception of the study and analysis: Judith Engels (JE), Johanna Kuske (JK), Kira Schmidt-Stiedenroth (KS), conducting the interviews: Johanna Kuske (JK), Rebekka Kuhlmann (RK), Anna Feisthauer (AF)
2.	Credentials	Judith Engels (M.A.), Johanna Kuske (M.Sc.), Kira Schmidt-Stiedenroth (PhD), Rebekka Kuhlmann (M.Sc.), Anna Feisthauer (Research assistant with Bachelor's degree)
3.	Occupation	JE, JK, KS, RK: research associates; AF: research assistant
4.	Gender	JE, JK, KS, RK, AF: female
5.	Experience and training	JE: educational background in business administration/psychology, practical experience in occupational health research, experience with qualitative research methods<br>JK: educational background in organizational psychology, practical experience in occupational health research, experience with qualitative research methods<br>KS: educational background in anthropology and public health, practical experience in occupational health research, experience with qualitative research methods<br>RK: educational background in psychology, practical experience in occupational health research<br>AF: educational background in psychology and medicine, practical experience in occupational health research
Relationship with participants		
6.	Relationship established	No
7.	Participant knowledge of interviewer	Participants knew JK, RK, AF as researchers from preliminary contact prior to the interviews
8.	Interviewer characteristics	No other characteristics were reported about the interviewer
**Domain 2: Study design**		
Theoretical framework		
9.	Methodological orientation and theory	Qualitative content analysis of Kuckartz (Kuckartz, 2018)
Participant selection		
10.	Sampling	Convenience and snowball sampling
11.	Method of approach	Face-to-face, e-mail
12.	Sample size	12 participants
13.	Non-participation	Not applicable
Setting		
14.	Setting of data collection	Participants participated in interviews online via a web conferencing software
15.	Presence of non-participants	No
16.	Description of sample	Managers between 34 and 62 years (M = 49), 2 males, 4 females; intercorporate stakeholders between 36 and 60 years (M=44), 4 males, 2 females
Data collection		
17.	Interview guide	Provided as supplemental material; Interview guide was piloted before study
18.	Repeat interviews	None
19.	Audio/visual recording	Audio recording
20.	Field notes	None
21.	Duration	27 – 50 minutes (managers), 24 – 68 minutes (intercorporate stakeholders)
22.	Data saturation	Yes
23.	Transcripts returned	No
**Domain 3: Analysis and findings**		
Data analysis		
24.	Number of data coders	Three (JE, JK, KS)
25.	Description of coding tree	No
26.	Derivation of themes	Deductive coding examples: acceptability; appropriateness, feasibility
27.	Software	MAXQDA 2020
28.	Participant checking	No
Reporting		
29.	Quotations presented	Yes
30.	Data and findings consistent	Yes
31.	Clarity of major themes	Yes
32.	Clarity of minor themes	Yes

Developed from: Tong A, Sainsbury P, Craig J. Consolidated criteria for reporting qualitative research (COREQ): a 32-item checklist for interviews and focus groups. International Journal for Quality in Health Care. 2007. Volume 19, Number 6: pp. 349 – 357
